# Supplementary material for: Design and Synthesis of Pyrrolidinyl Ferrocene-Containing Ligands and Their Application in Highly Enantioselective Rhodium-Catalyzed Olefin Hydrogenation
Source: Molecules. 2022 Sep 17;27(18):6078. doi: 10.3390/molecules27186078 (PMC9505515; doi:10.3390/molecules27186078)
Supplement: Supplementary file 1 [file molecules-27-06078-s001.zip › molecules-1889876-supplementary.pdf]

Design and Synthesis of Pyrrolidinyl Ferrocene-containing Ligands and Their Application in Highly Enantioselective Rhodium-catalyzed Olefin Hydrogenation

Xin Li, Cian Kingston, Yannick Ortin and Patrick J. Guiry*

Supplementary Information

Table of Contents

Methods for the Determination of Enantiomeric Excess (Table S1) ……...………………...…… 2

SFC Chromatograms of Racemic and Enantioenriched Compounds……..…………….…….………… 4

^{1}H NMR, ^{13}C NMR and ^{31}P NMR Spectra of New Compounds.……………………..….…...... 21

X-Ray Crystallographic Structure for **L5**.…………………….…....... 49

Methods for the Determination of Enantiomeric Excess (Table S1)

Entry	Product	Assay Conditions	Retention time (min) of major enantiomer	Retention time (min) of minor enantiomer
1	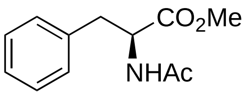 **17a**	HPLC, Chiralcel AD column, Isocratic 90/10 IPA/*n*-hex, 25 °C oven, 1 mL/min flow, 220nm	11.59	9.07
2	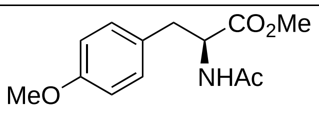 **17b**	HPLC, Chiralcel AD column, Isocratic 85/15 IPA/*n*-hex, 25 °C oven, 1 mL/min flow, 220nm	10.47	8.14
3	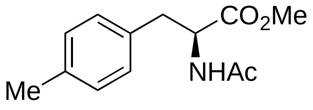 **17c**	HPLC, Chiralcel AD column, Isocratic 90/10 IPA/*n*-hex, 25 °C oven, 1 mL/min flow, 220nm	11.68	9.07
4	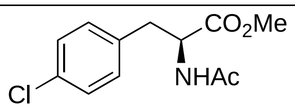 **17d**	HPLC, Chiralcel AD column, Isocratic 90/10 IPA/*n*-hex, 25 °C oven, 1 mL/min flow, 220nm	12.44	10.47
5	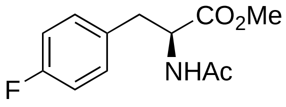 **17e**	HPLC, Chiralcel AD column, Isocratic 90/10 IPA/*n*-hex, 25 °C oven, 1 mL/min flow, 220nm	12.58	10.88
6	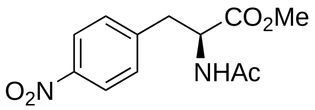 **17f**	HPLC, Chiralcel AD column, Isocratic 90/10 IPA/*n*-hex, 25 °C oven, 1 mL/min flow, 254nm	29.97	27.33
7	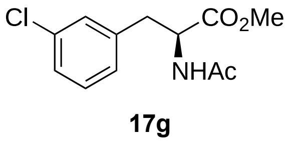 **17g**	HPLC, Chiralcel AD column, Isocratic 90/10 IPA/*n*-hex, 25 °C oven, 1 mL/min flow, 220nm	10.42	8.89
8	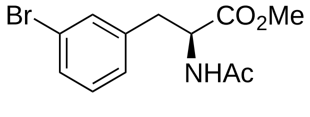 **17h**	HPLC, Chiralcel OD column, Isocratic 95/5 IPA/*n*-hex, 25 °C oven, 1 mL/min flow, 220nm	24.03	21.02
9	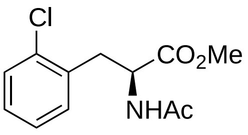 **17i**	HPLC, Chiralcel AD column, Isocratic 90/10 IPA/*n*-hex, 25 °C oven, 1 mL/min flow, 220nm	12.58	9.41

10	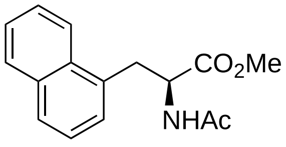<br>**17j**	HPLC, Chiralcel OD column, Isocratic 90/10 IPA/*n*-hex, 25 °C oven, 1 mL/min flow, 220nm	13.25	10.53
11	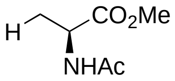<br>**17k**	HPLC, Chiralcel AD column, Isocratic 93/7 IPA/*n*-hex, 25 °C oven, 1 mL/min flow, 210nm	10.83	9.45
12	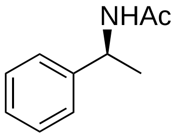<br>**19a**	HPLC, Chiralcel AD column, Isocratic 94/6 IPA/*n*-hex, 25 °C oven, 1 mL/min flow, 220nm	11.63	9.72
13	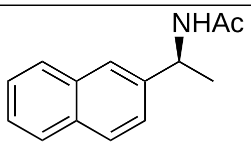<br>**19b**	HPLC, Chiralcel AD column, Isocratic 90/10 IPA/*n*-hex, 25 °C oven, 1 mL/min flow, 254nm	9.95	7.63
14	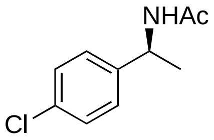<br>**19c**	HPLC, Chiralcel AD column, Isocratic 90/10 IPA/*n*-hex, 25 °C oven, 1 mL/min flow, 254nm	11.13	9.28
15	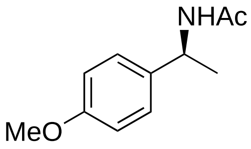<br>**19d**	HPLC, Chiralcel AD column, Isocratic 93/7 IPA/*n*-hex, 25 °C oven, 1 mL/min flow, 220nm	15.18	12.57
16	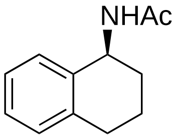<br>**19e**	HPLC, Chiralcel OD column, Isocratic 90/10 IPA/*n*-hex, 25 °C oven, 1 mL/min flow, 210nm	9.50	12.83
17	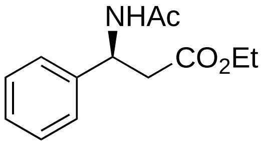<br>**19f**	HPLC, Chiralcel OD column, Isocratic 95/5 IPA/*n*-hex, 25 °C oven, 1 mL/min flow, 220nm	30.91	25.78

SFC Chromatograms of Racemic and Enantioenriched Compounds

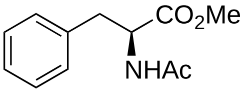

17a

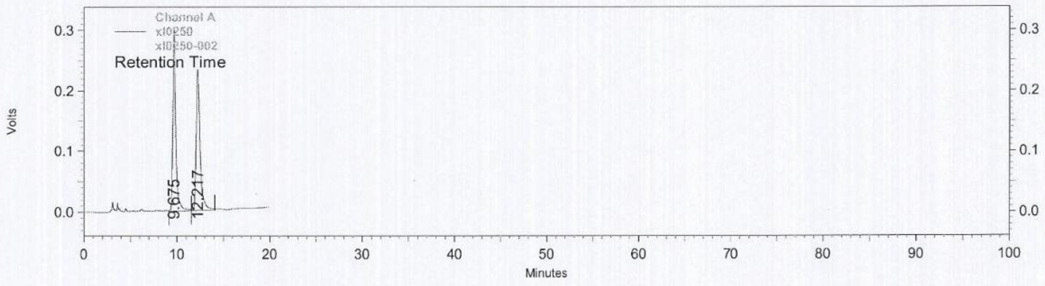

Detector A (220nm)

Pk #	Retention Time	Area	Area %	Height	Height %
1	9.675	6698945	49.900	299214	56.231
2	12.217	6725775	50.100	232903	43.769
Totals		13424720	100.000	532117	100.000

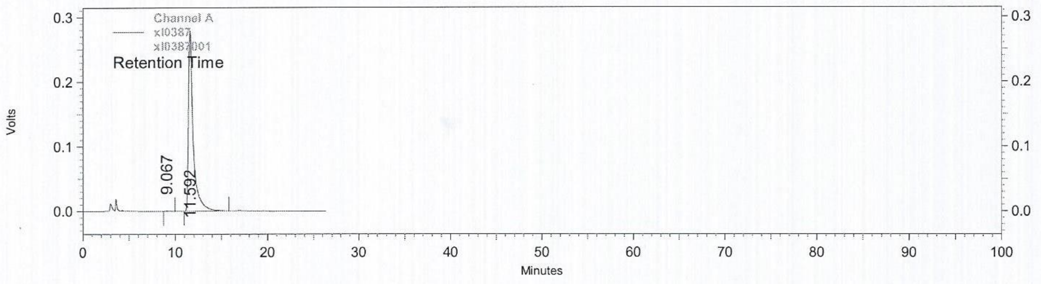

Detector A (220nm)

Pk #	Retention Time	Area	Area %	Height	Height %
1	9.067	26854	0.266	962	0.343
2	11.592	10051910	99.734	279605	99.657
Totals		10078764	100.000	280567	100.000

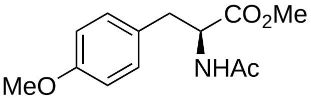

17b

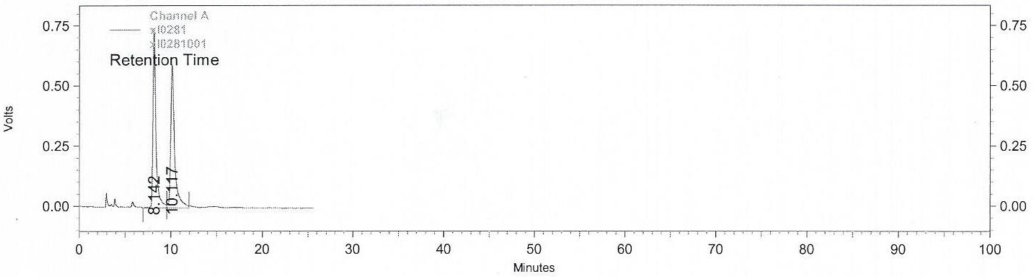

Detector A (220nm)

Pk #	Retention Time	Area	Area %	Height	Height %
1	8.142	16695674	48.577	747180	55.722
2	10.117	17673937	51.423	593719	44.278
Totals		34369611	100.000	1340899	100.000

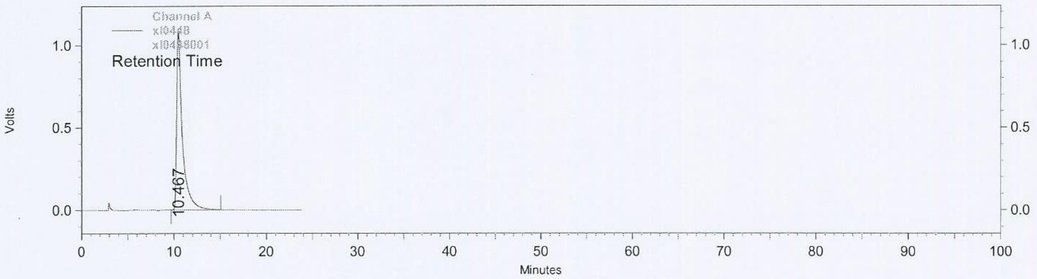

Detector A (220nm)

Pk #	Retention Time	Area	Area %	Height	Height %
1	10.467	46948277	100.000	1099114	100.000
Totals		46948277	100.000	1099114	100.000

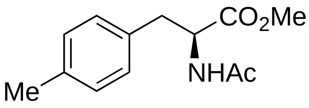

17c

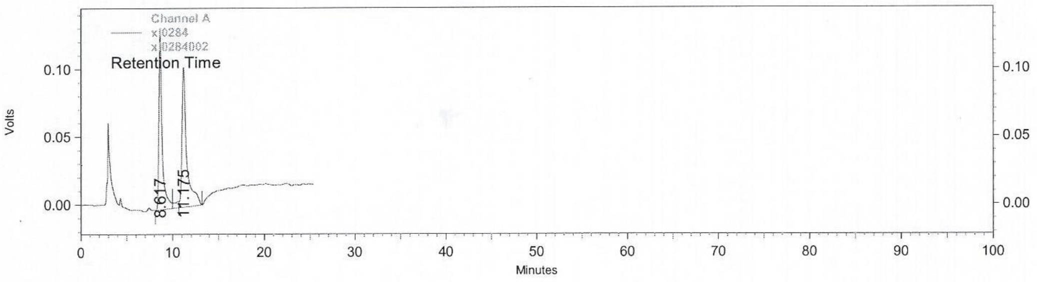

Detector A (220nm)

Pk #	Retention Time	Area	Area %	Height	Height %
1	8.617	3224503	46.140	132105	56.177
2	11.175	3764000	53.860	103054	43.823
Totals		6988503	100.000	235159	100.000

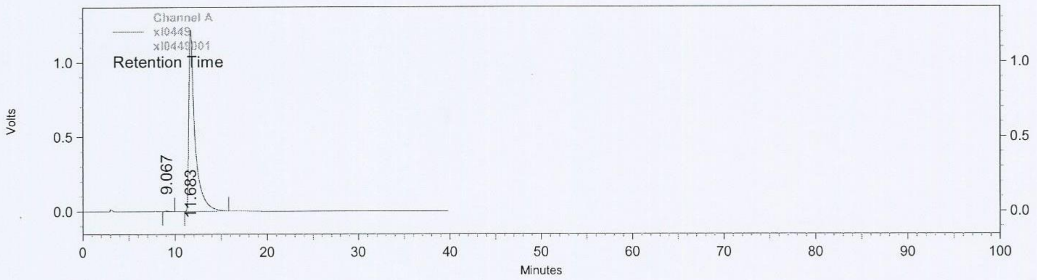

Detector A (220nm)

Pk #	Retention Time	Area	Area %	Height	Height %
1	9.067	168652	0.299	5646	0.461
2	11.683	56254004	99.701	1218756	99.539
Totals		56422656	100.000	1224402	100.000

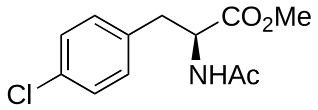

17d

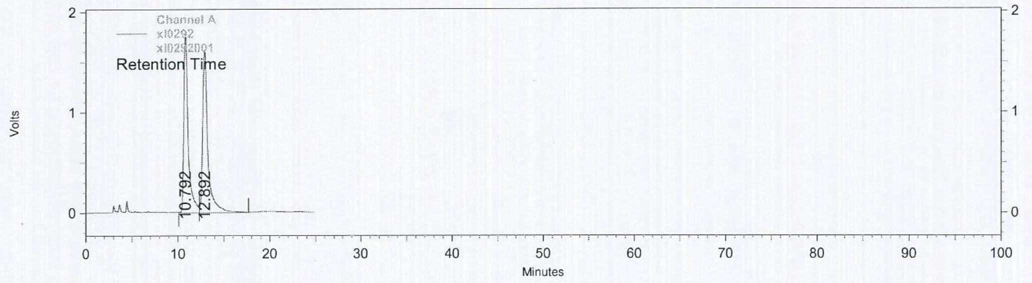

Detector A (220nm)

Pk #	Retention Time	Area	Area %	Height	Height %
1	10.792	59289906	47.913	1798402	52.546
2	12.892	64456210	52.087	1624154	47.454
Totals		123746116	100.000	3422556	100.000

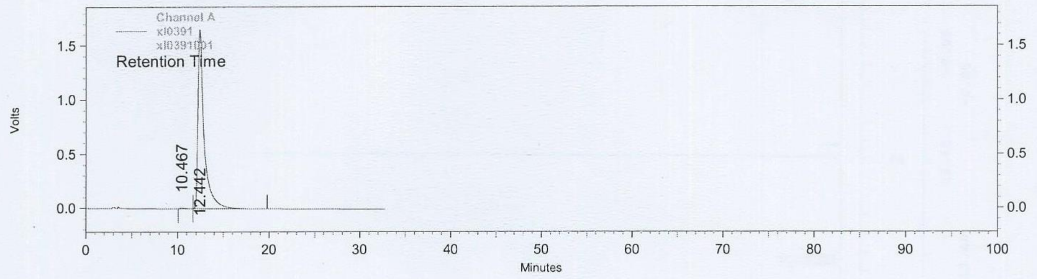

Detector A (220nm)

Pk #	Retention Time	Area	Area %	Height	Height %
1	10.467	221577	0.285	6516	0.392
2	12.442	77600571	99.715	1655071	99.608
Totals		77822148	100.000	1661587	100.000

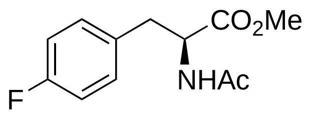

17e

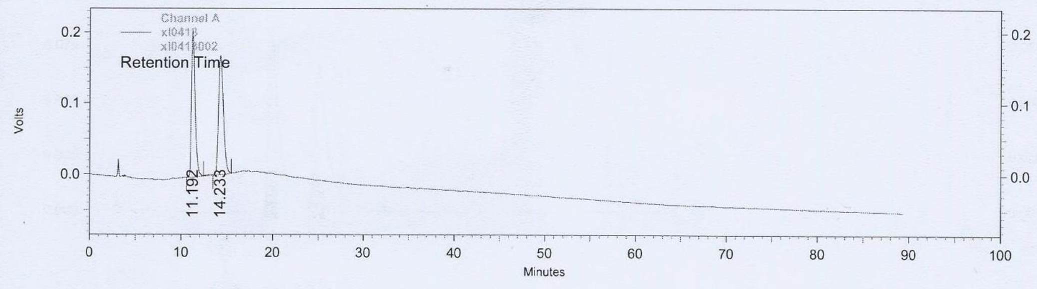

Detector A (220nm)

Pk #	Retention Time	Area	Area %	Height	Height %
1	11.192	5755130	48.120	206315	55.196
2	14.233	6204842	51.880	167472	44.804
Totals		11959972	100.000	373787	100.000

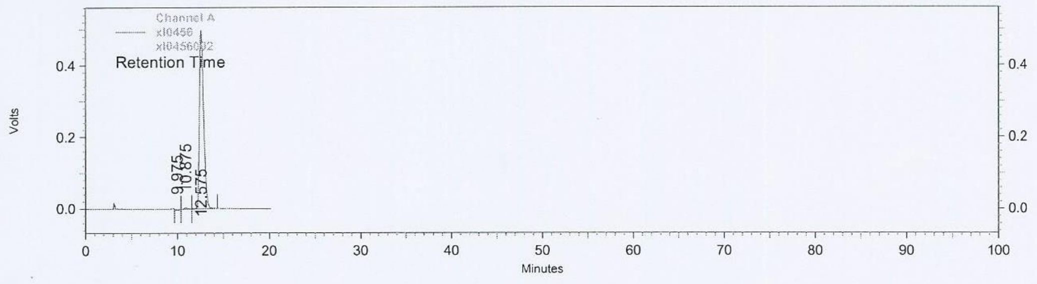

Detector A (220nm)

Pk #	Retention Time	Area	Area %	Height	Height %
1	9.975	63140	0.395	2903	0.576
2	10.875	122709	0.768	3681	0.730
3	12.575	15797125	98.837	497357	98.693
Totals		15982974	100.000	503941	100.000

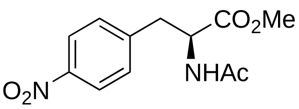

17f

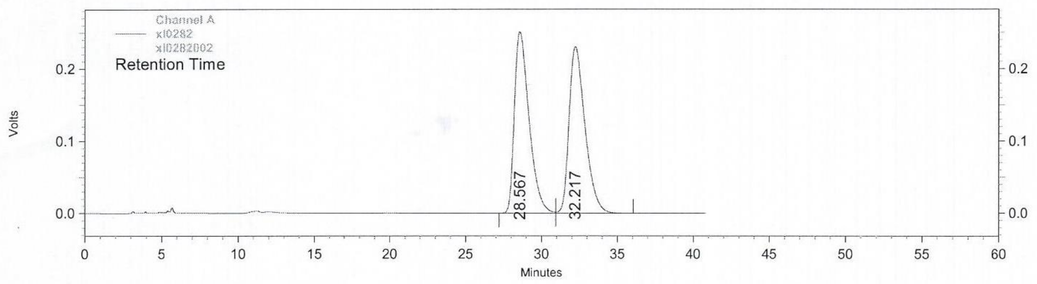

Detector A (254nm)

Pk #	Retention Time	Area	Area %	Height	Height %
1	28.567	16899144	49.918	251033	52.164
2	32.217	16954378	50.082	230205	47.836
Totals		33853522	100.000	481238	100.000

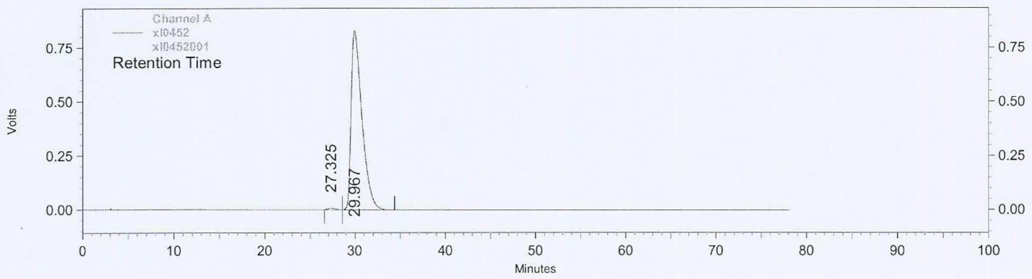

Detector A (254nm)

Pk #	Retention Time	Area	Area %	Height	Height %
1	27.325	499490	0.716	7756	0.925
2	29.967	69265058	99.284	830569	99.075
Totals		69764548	100.000	838325	100.000

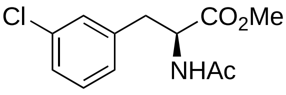

17g

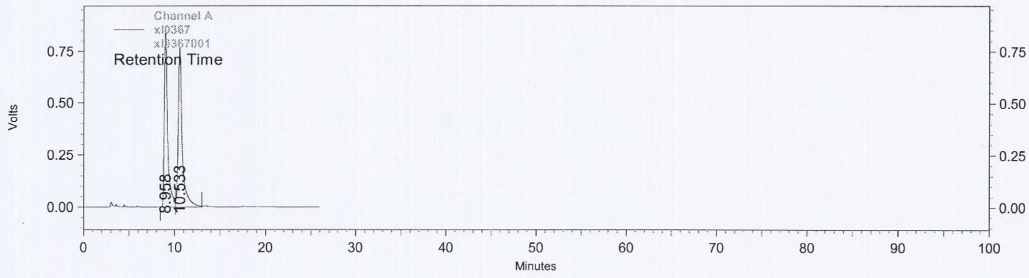

Detector A (220nm)

Pk #	Retention Time	Area	Area %	Height	Height %
1	8.958	21923789	48.043	861946	52.980
2	10.533	23710224	51.957	764995	47.020
Totals		45634013	100.000	1626941	100.000

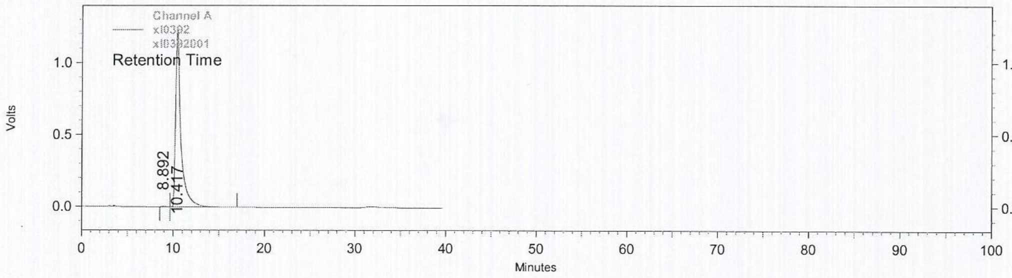

Detector A (220nm)

Pk #	Retention Time	Area	Area %	Height	Height %
1	8.892	138584	0.276	4925	0.395
2	10.417	49997588	99.724	1242624	99.605
Totals		50136172	100.000	1247549	100.000

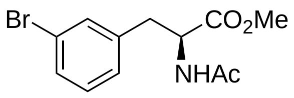

17h

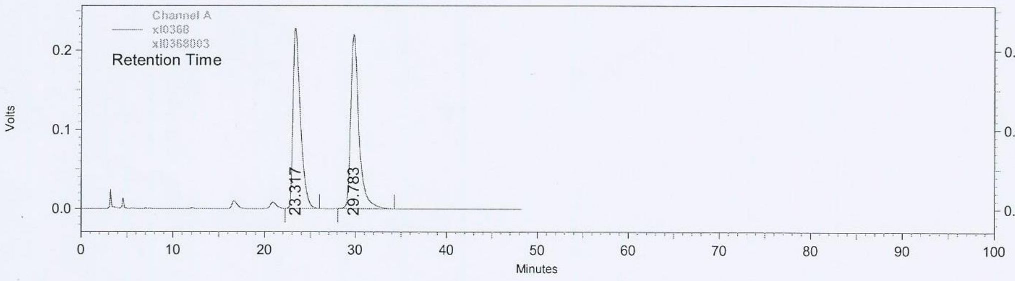

Detector A (220nm)

Pk #	Retention Time	Area	Area %	Height	Height %
1	23.317	12842045	48.218	227695	50.932
2	29.783	13791122	51.782	219366	49.068
Totals		26633167	100.000	447061	100.000

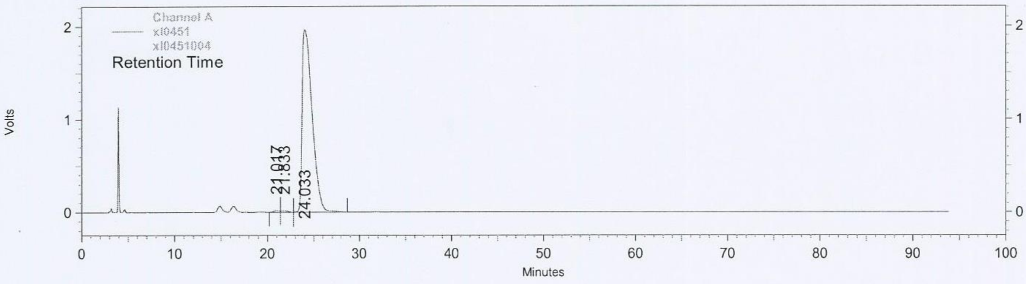

Detector A (220nm)

Pk #	Retention Time	Area	Area %	Height	Height %
1	21.017	726459	0.486	17448	0.873
2	21.833	717214	0.480	15123	0.756
3	24.033	148073711	99.034	1966872	98.371
Totals		149517384	100.000	1999443	100.000

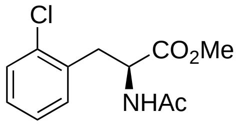

17i

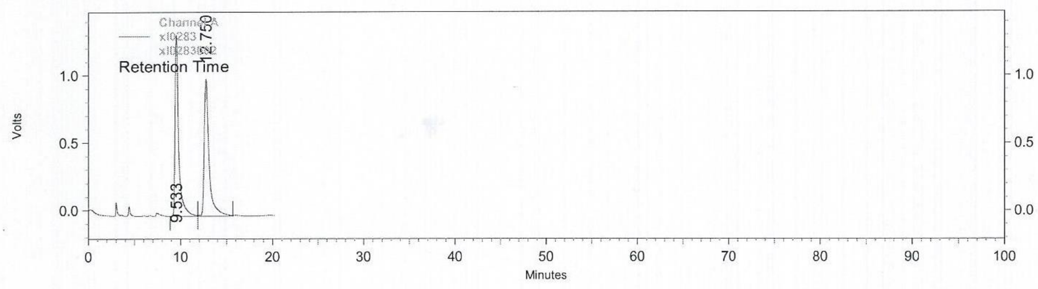

Detector A (220nm)

Pk #	Retention Time	Area	Area %	Height	Height %
1	9.533	36998543	49.678	1346372	57.003
2	12.750	37478706	50.322	1015557	42.997
Totals		74477249	100.000	2361929	100.000

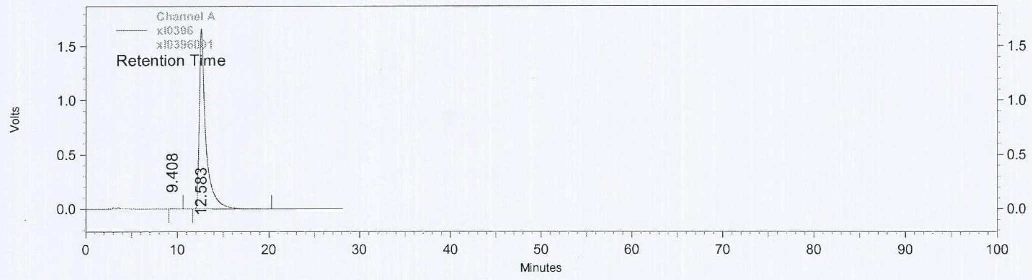

Detector A (220nm)

Pk #	Retention Time	Area	Area %	Height	Height %
1	9.408	130207	0.159	4008	0.240
2	12.583	81890223	99.841	1664224	99.760
Totals		82020430	100.000	1668232	100.000

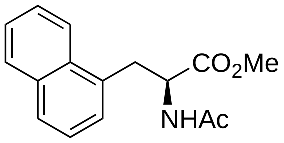

17j

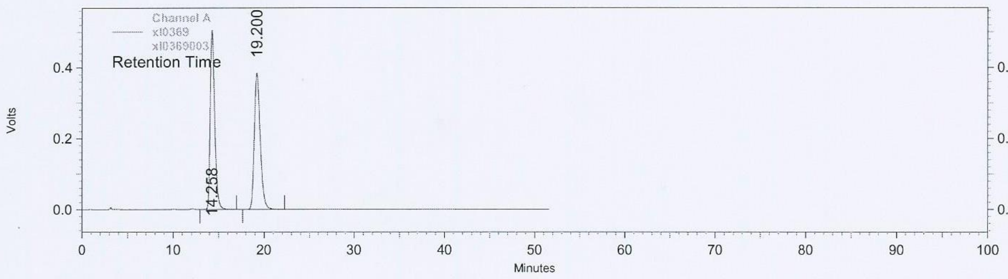

Detector A (220nm)

Pk #	Retention Time	Area	Area %	Height	Height %
1	14.258	17461334	49.827	505483	56.831
2	19.200	17582931	50.173	383966	43.169
Totals		35044265	100.000	889449	100.000

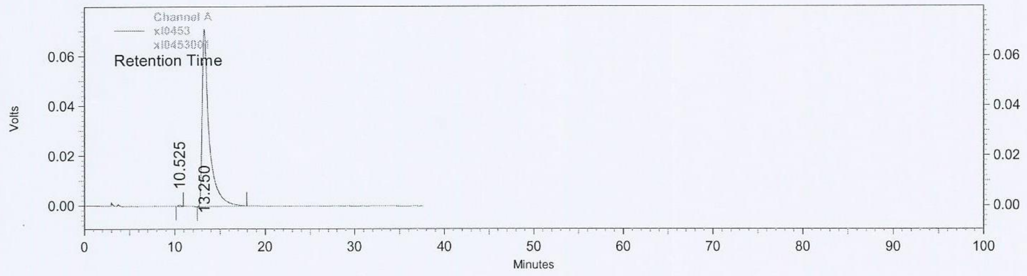

Detector A (254nm)

Pk #	Retention Time	Area	Area %	Height	Height %
1	10.525	16279	0.423	577	0.802
2	13.250	3827775	99.577	71342	99.198
Totals		3844054	100.000	71919	100.000

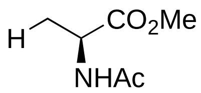

17k

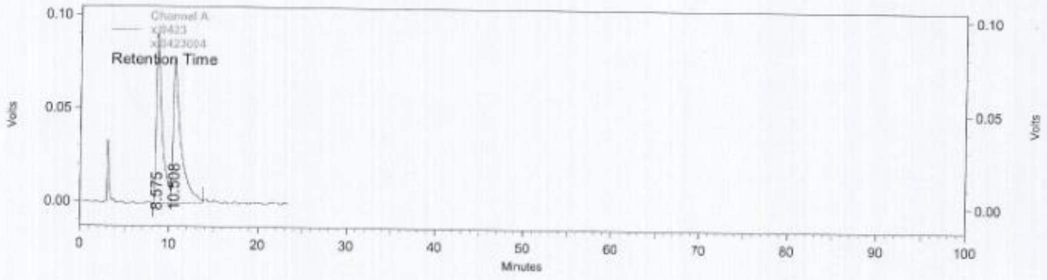

Detector A (210nm)

Pk #	Retention Time	Area	Area %	Height	Height %
1	8.575	4158036	46.888	93808	54.947
2	10.508	4710061	53.112	76916	45.053
Totals		8868097	100.000	170724	100.000

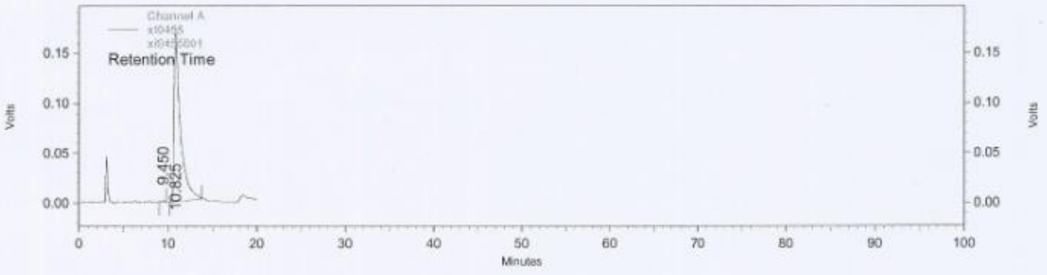

Detector A (210nm)

Pk #	Retention Time	Area	Area %	Height	Height %
1	9.450	35480	0.425	1241	0.711
2	10.825	8321823	99.575	173218	99.289
Totals		8357303	100.000	174459	100.000

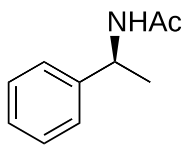

19a

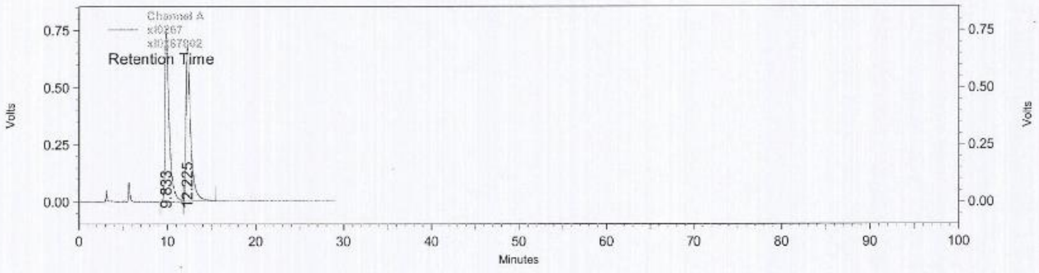

Detector A (220nm)

Pk #	Retention Time	Area	Area %	Height	Height %
1	9.833	24847348	49.782	759934	52.739
2	12.225	25065435	50.218	681002	47.261
Totals		49912783	100.000	1440936	100.000

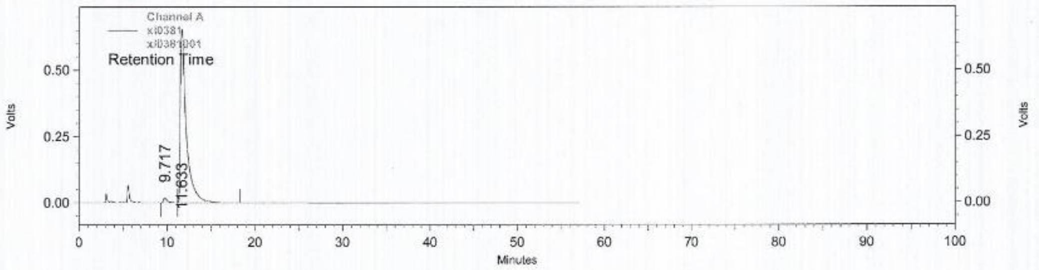

Detector A (220nm)

Pk #	Retention Time	Area	Area %	Height	Height %
1	9.717	565978	1.791	18055	2.672
2	11.633	31043172	98.209	657640	97.328
Totals		31609150	100.000	675695	100.000

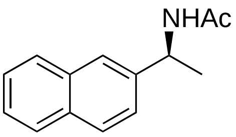

19b

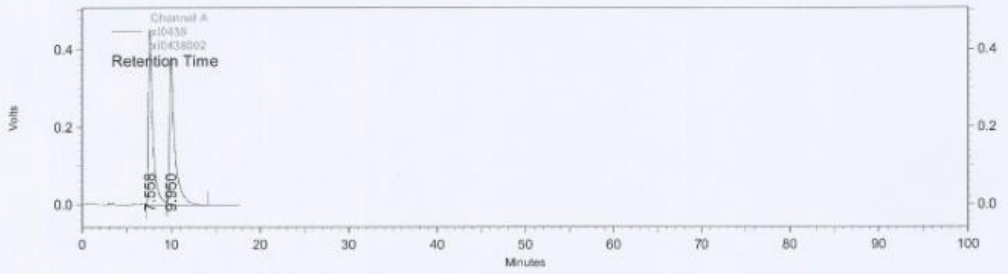

Detector A (254nm)

Pk #	Retention Time	Area	Area %	Height	Height %
1	7.558	15364732	49.626	451099	54.095
2	9.950	15596571	50.374	382803	45.905
Totals		30961303	100.000	833902	100.000

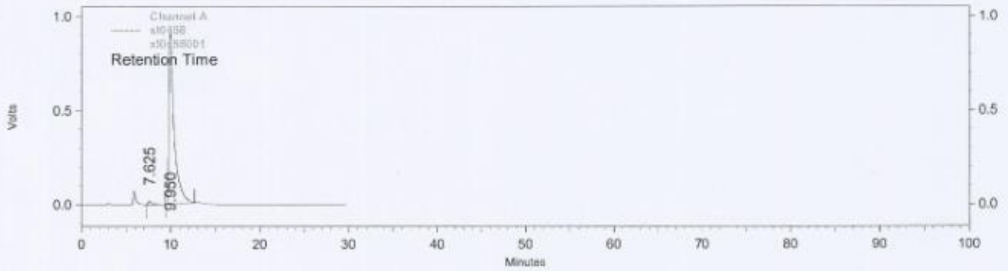

Detector A (254nm)

Pk #	Retention Time	Area	Area %	Height	Height %
1	7.625	500478	1.309	17588	1.850
2	9.950	37728825	98.691	933308	98.150
Totals		38229303	100.000	950896	100.000

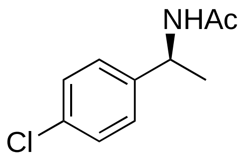

19c

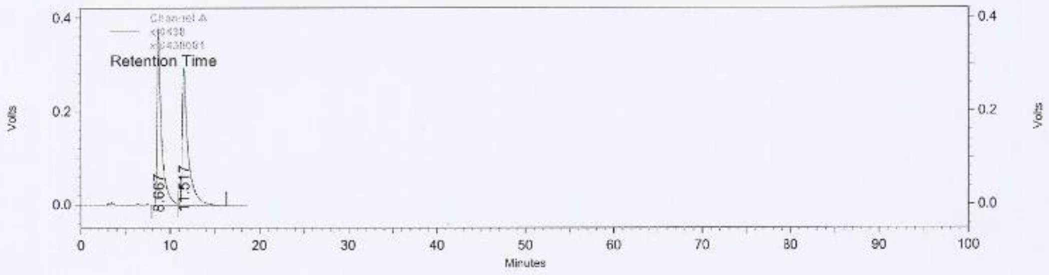

Detector A (254nm)

Pk #	Retention Time	Area	Area %	Height	Height %
1	8.667	13432742	49.586	375088	56.105
2	11.517	13657216	50.414	293462	43.895
Totals		27089958	100.000	668550	100.000

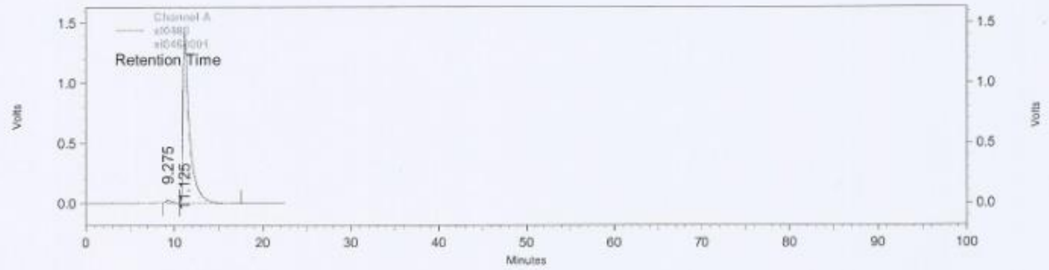

Detector A (220nm)

Pk #	Retention Time	Area	Area %	Height	Height %
1	9.275	866539	1.186	24482	1.666
2	11.125	72205768	98.814	1444973	98.334
Totals		73072307	100.000	1469455	100.000

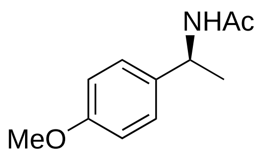

19d

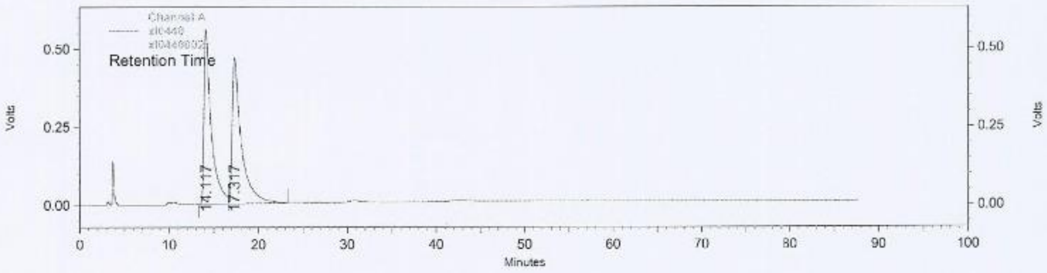

Detector A (220nm)

Pk #	Retention Time	Area	Area %	Height	Height %
1	14.117	31342228	48.488	557655	54.482
2	17.317	33297387	51.512	465897	45.518
Totals		64639615	100.000	1023552	100.000

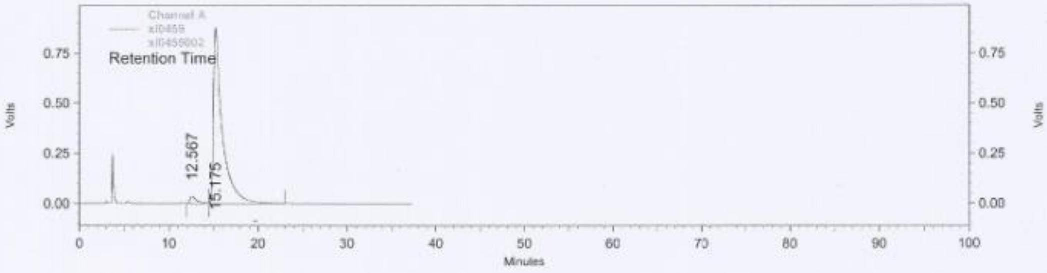

Detector A (220nm)

Pk #	Retention Time	Area	Area %	Height	Height %
1	12.567	1971086	3.119	36448	3.979
2	15.175	61224732	96.881	879583	96.021
Totals		63195818	100.000	916031	100.000

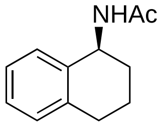

19e

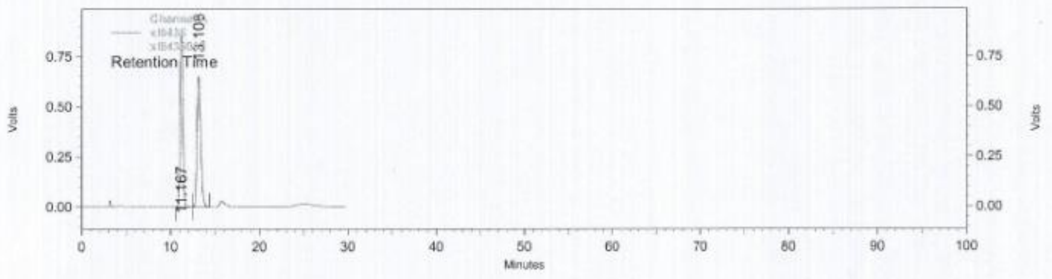

Detector A (210nm)

Pk #	Retention Time	Area	Area %	Height	Height %
1	11.167	19010432	49.648	878590	57.437
2	13.108	19280141	50.352	651060	42.563
Totals		38290573	100.000	1529650	100.000

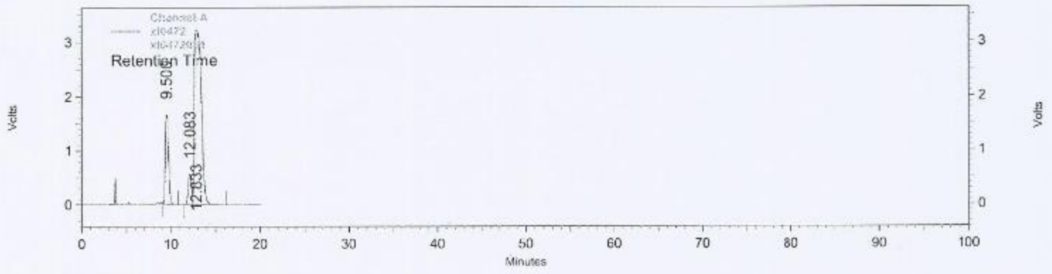

Detector A (210nm)

Pk #	Retention Time	Area	Area %	Height	Height %
1	9.500	41123301	17.967	1652152	30.338
2	12.083	14011210	6.122	563897	10.355
3	12.833	173748366	75.911	3229770	59.307
Totals		228882877	100.000	5445819	100.000

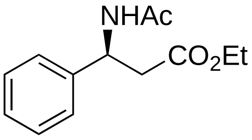

19f

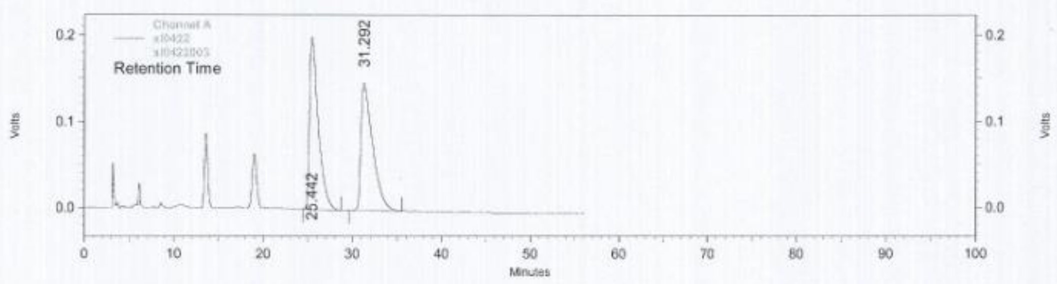

Detector A (220nm)

Pk #	Retention Time	Area	Area %	Height	Height %
1	25.442	13700804	50.240	199726	57.458
2	31.292	13570053	49.760	147880	42.542
Totals		27270857	100.000	347606	100.000

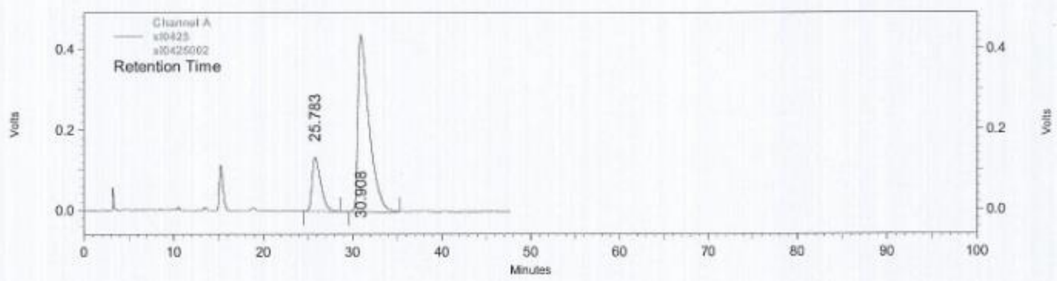

Detector A (210nm)

Pk #	Retention Time	Area	Area %	Height	Height %
1	25.783	9250656	19.320	135767	23.625
2	30.908	38629961	80.680	438919	76.375
Totals		47880617	100.000	574686	100.000

^{1}H NMR, ^{13}C NMR and ^{31}P NMR Spectra of New Compounds

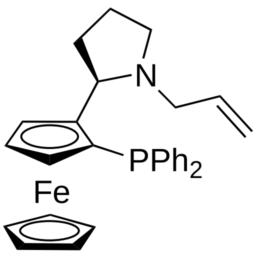

(*R*,*S*$_p$)-**14**

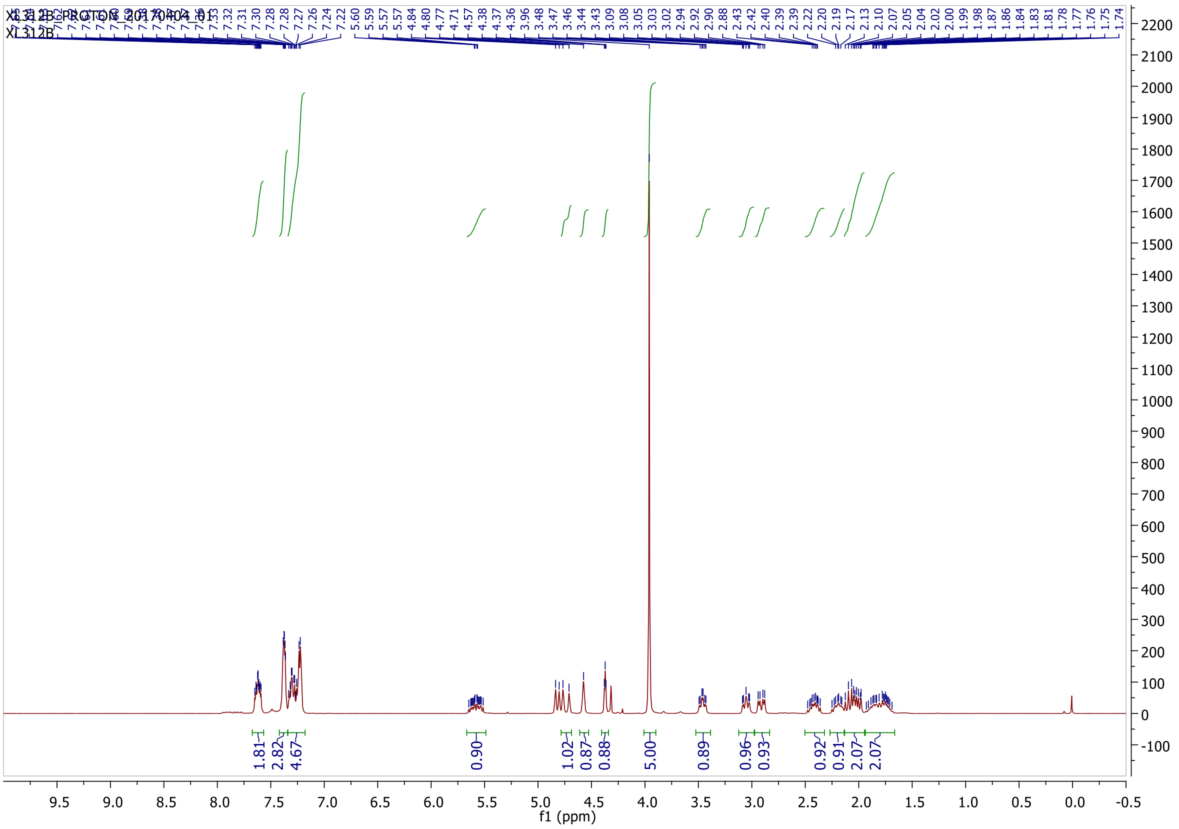

^{1}H NMR Spectrum of (*R*,*Sp*)-**14**

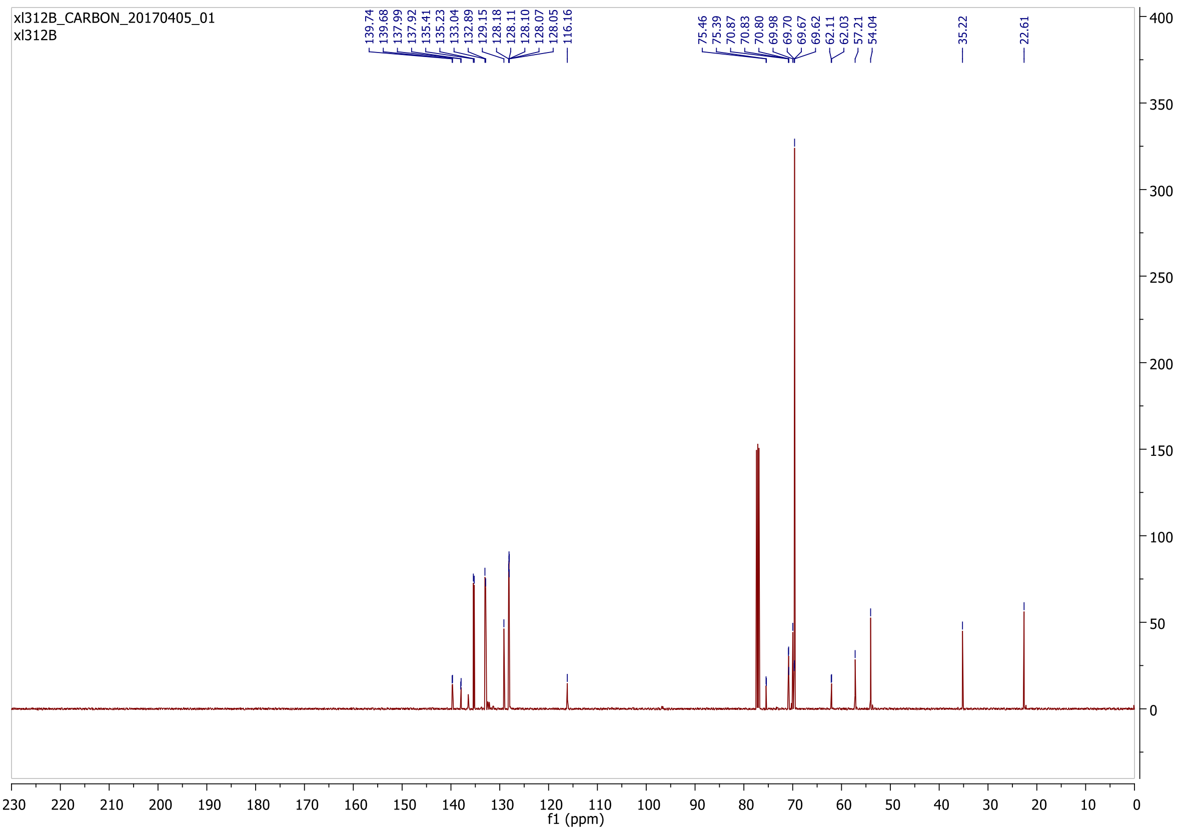

^{13}C NMR Spectrum of (*R*,*Sp*)-**14**

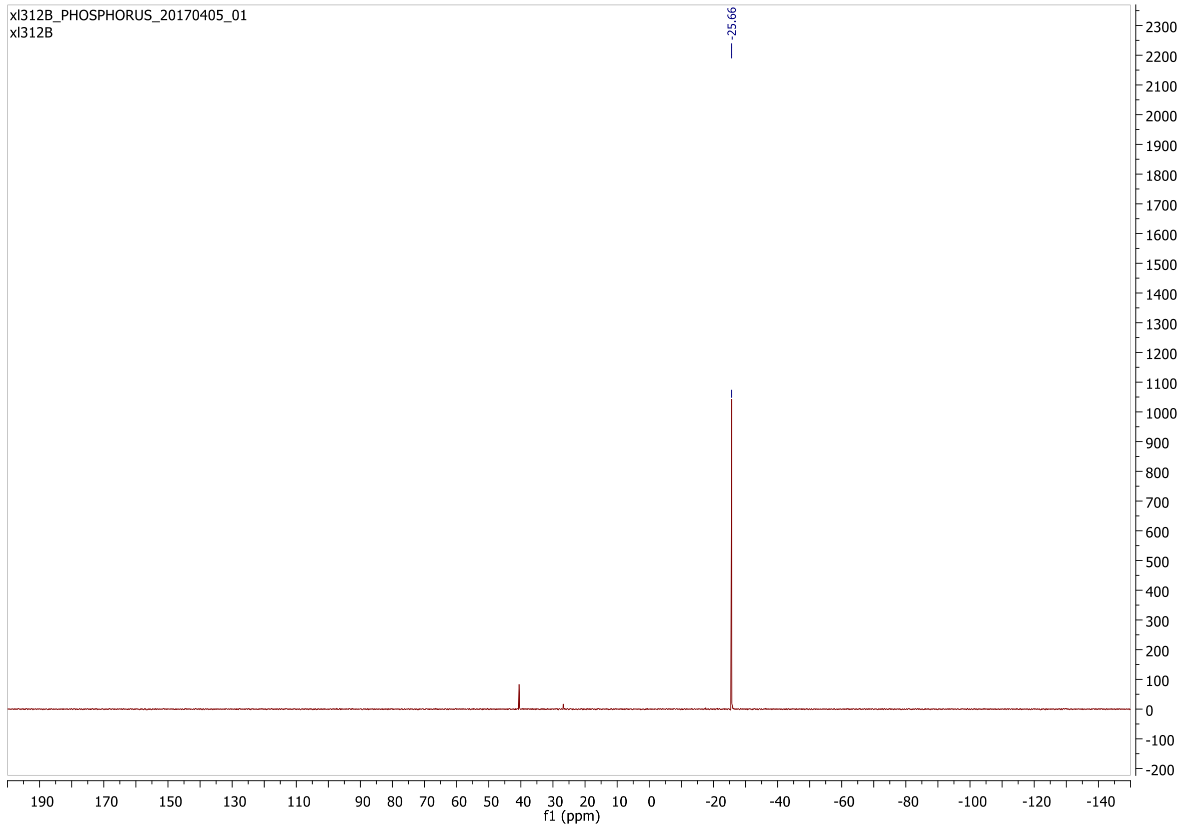

^{31}P NMR Spectrum of (*R*,*Sp*)-**14**

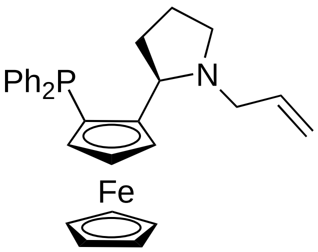

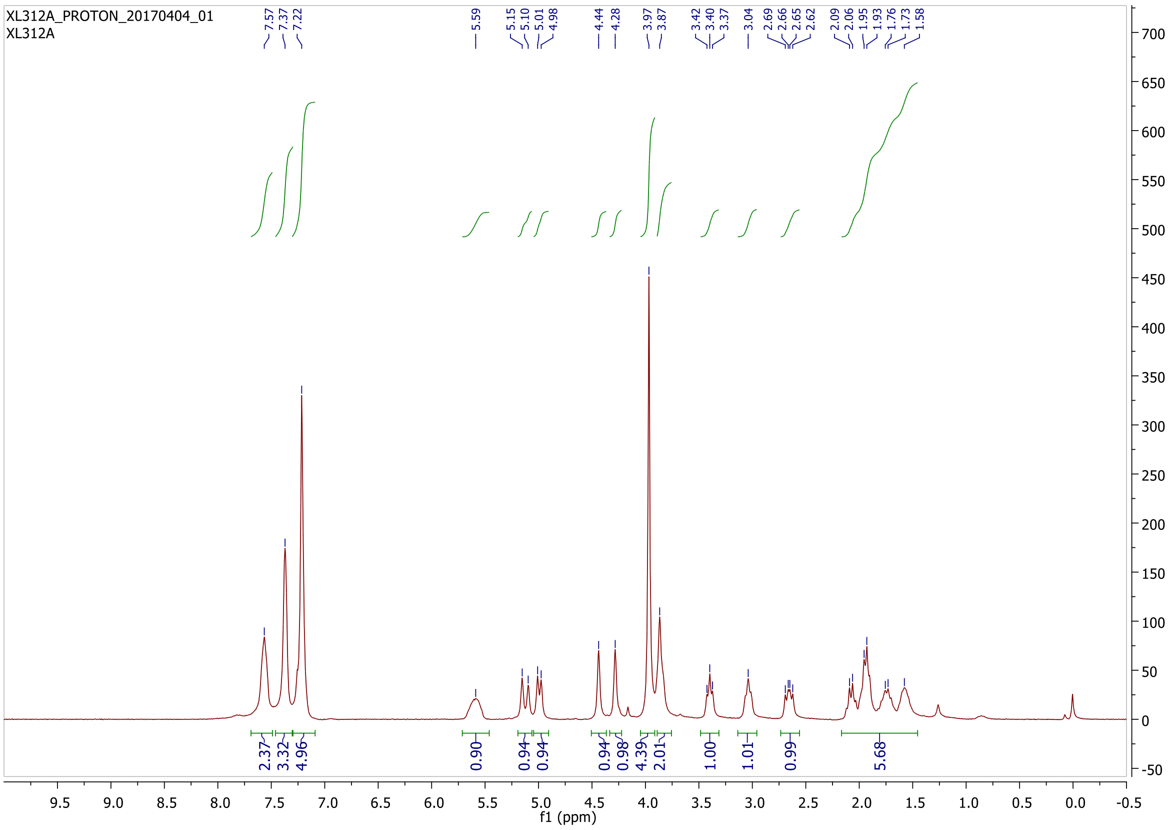

^{1}H NMR Spectrum of (*R*,*Rp*)-**14**

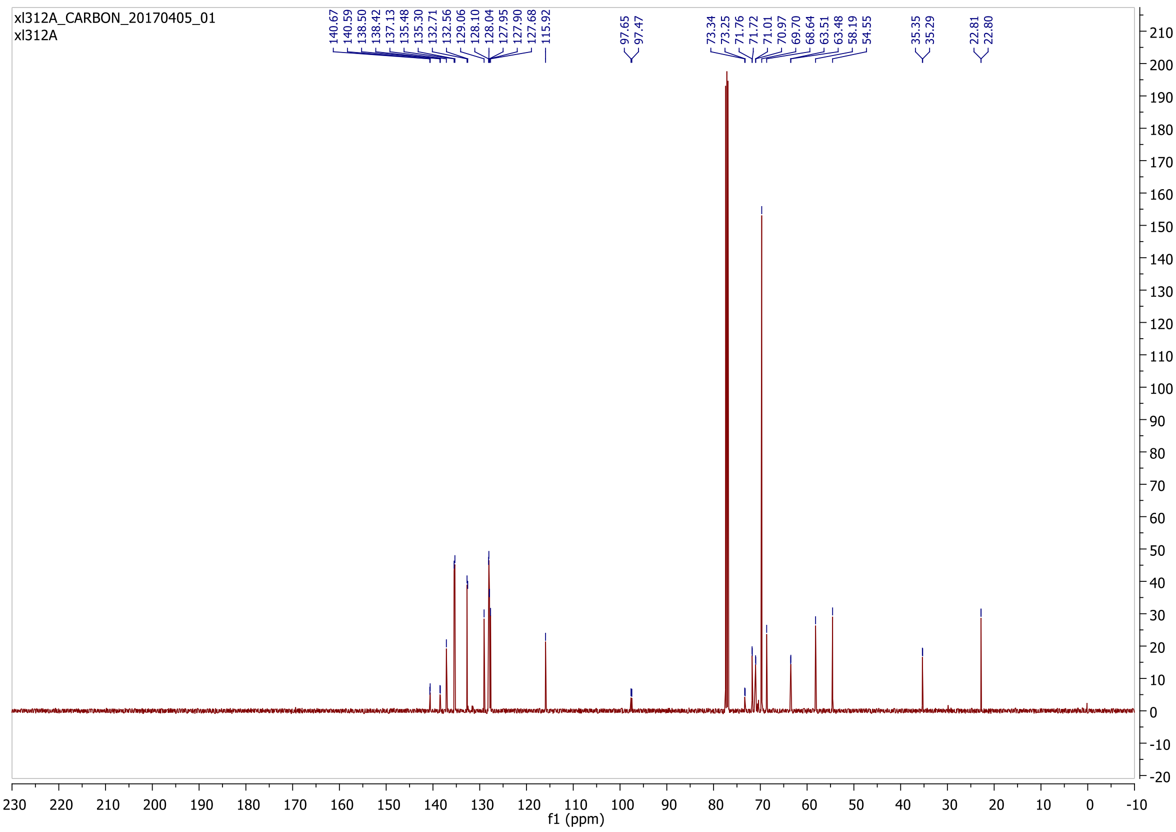

^{13}C NMR Spectrum of (*R*,*Rp*)-**14**

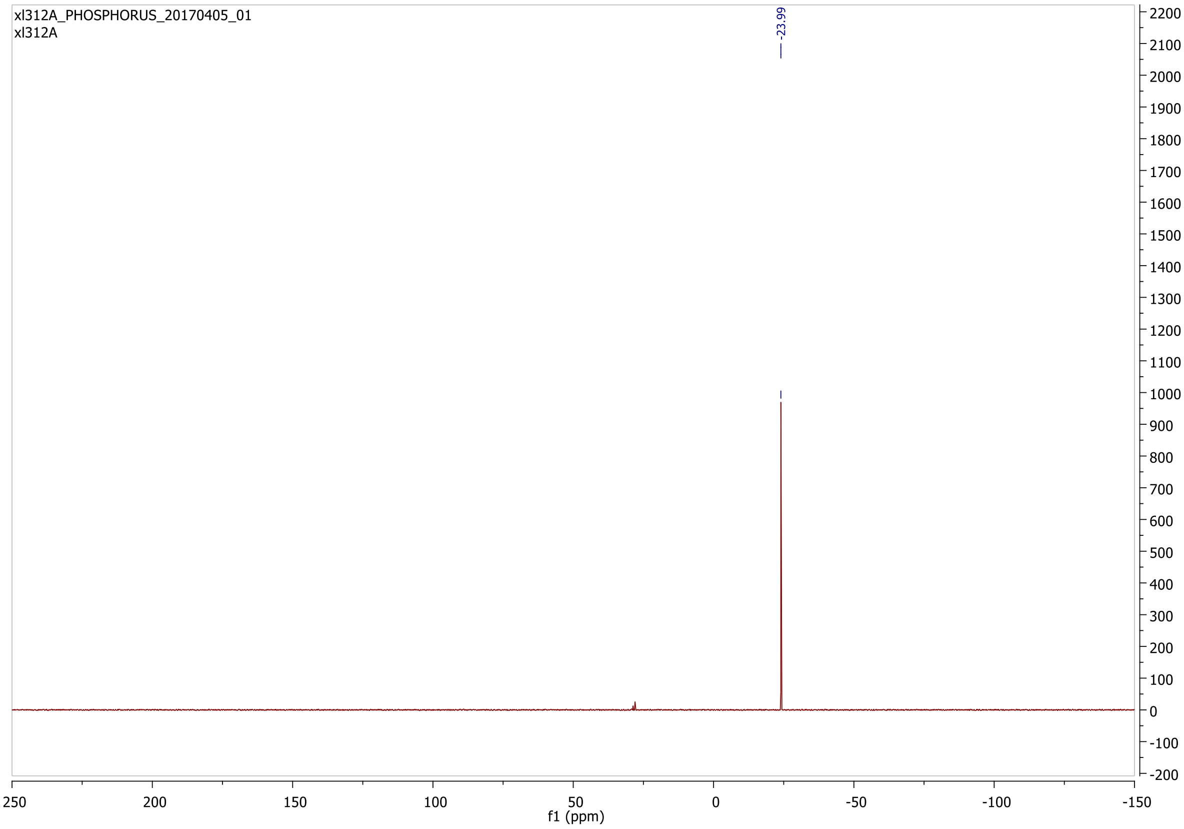

^{31}P NMR Spectrum of (*R*,*Rp*)-**14**

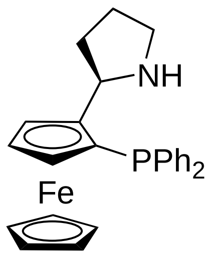

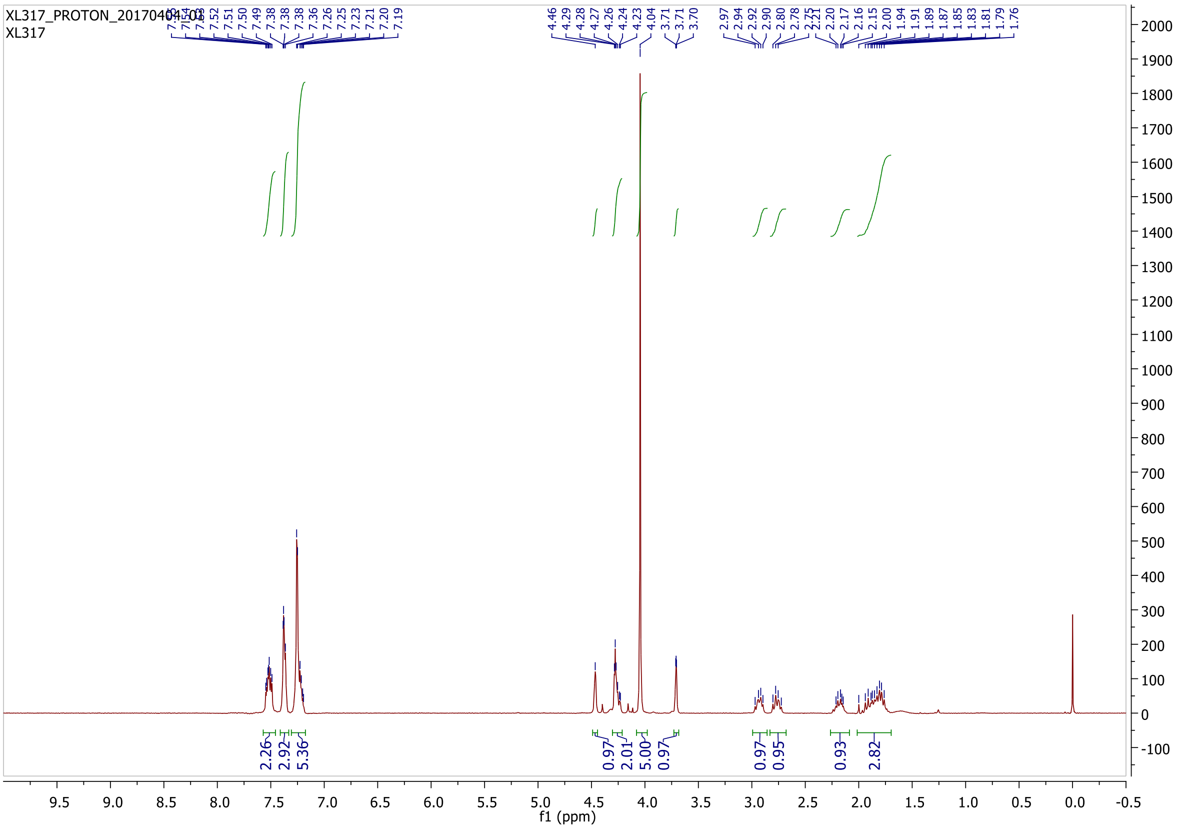

^{1}H NMR Spectrum of (*R*,*Sp*)-**15**

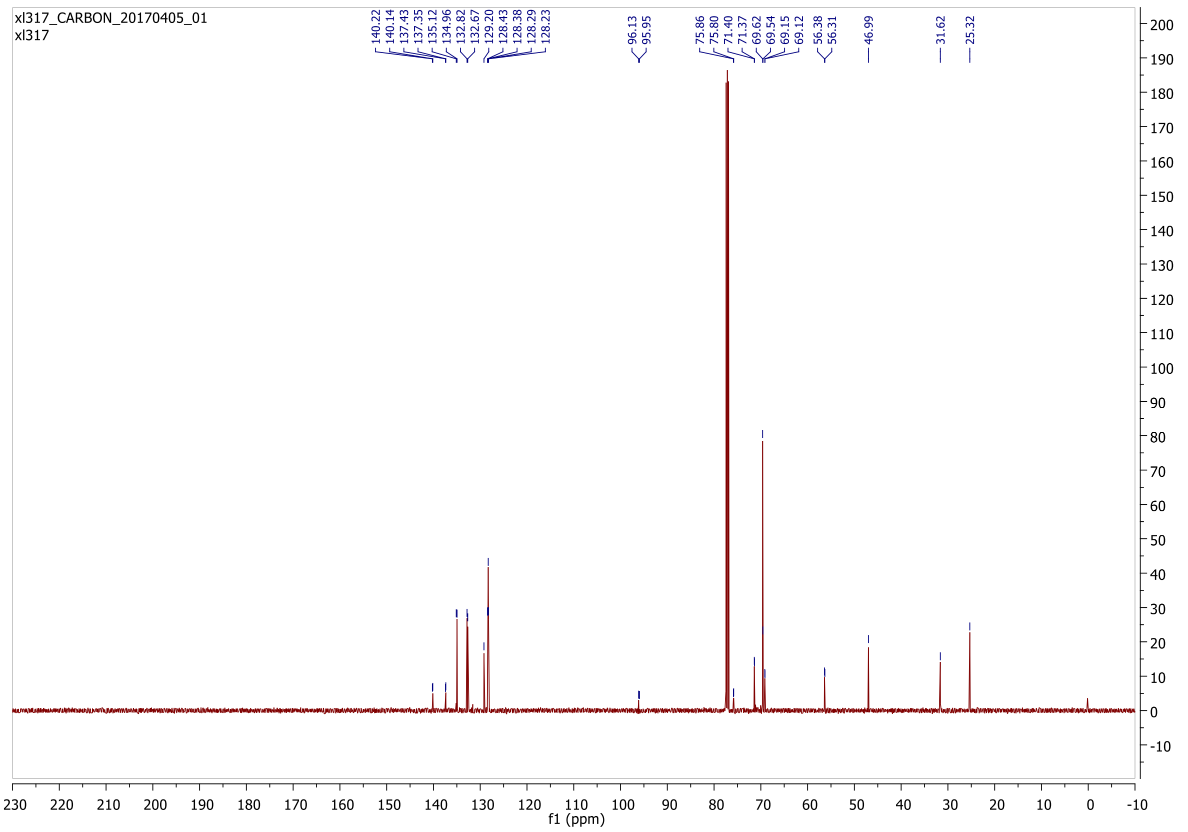

^{13}C NMR Spectrum of (*R*,*Sp*)-**15**

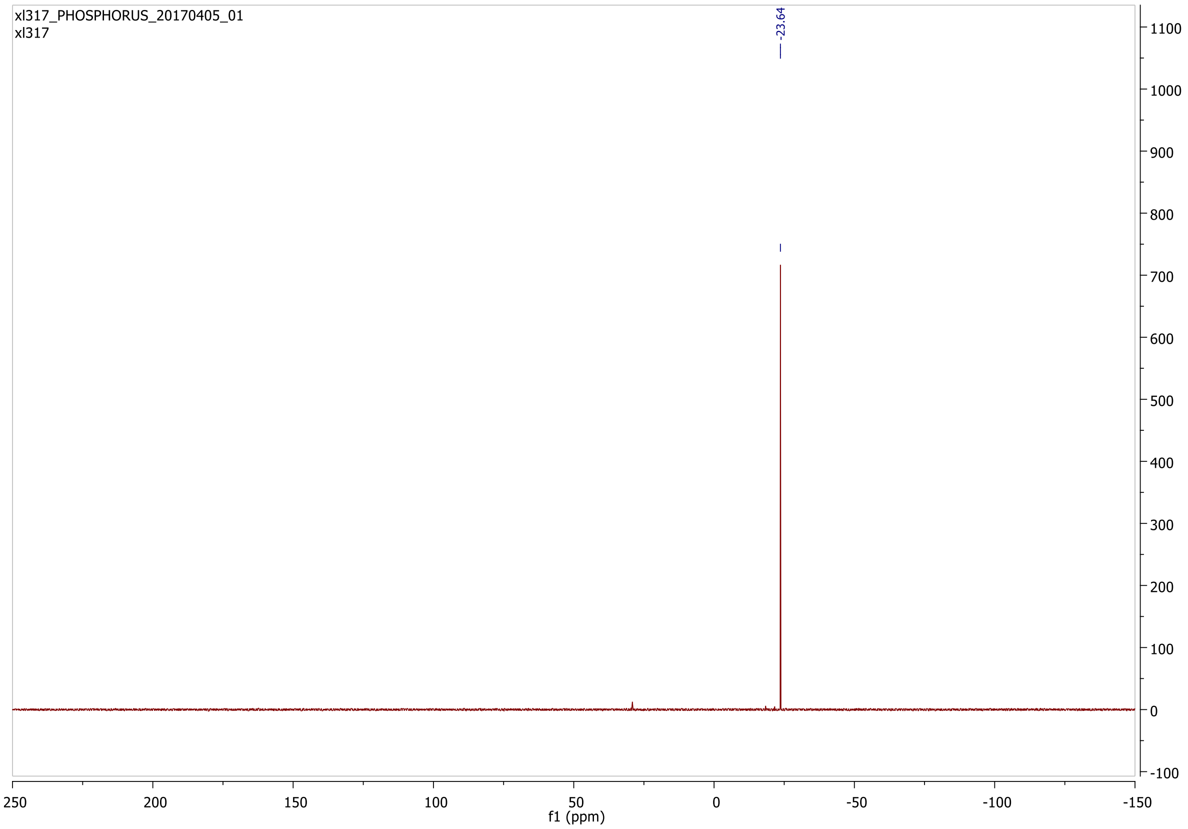

^{31}P NMR Spectrum of (*R*,*Sp*)-**15**

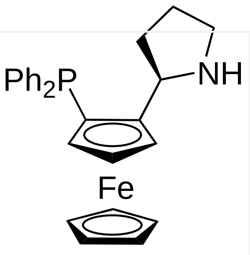

(*R*,*R*$_p$)-**15**

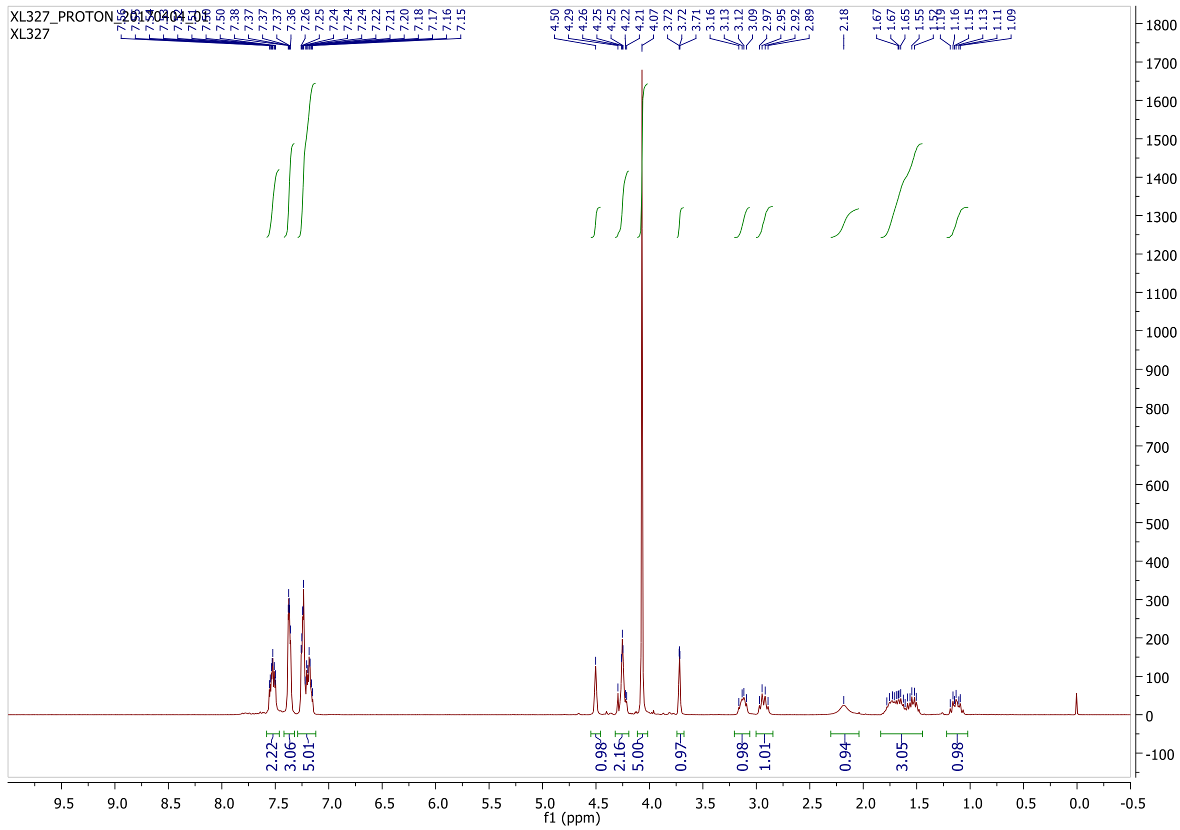

^{1}H NMR Spectrum of (*R*,*Rp*)-**15**

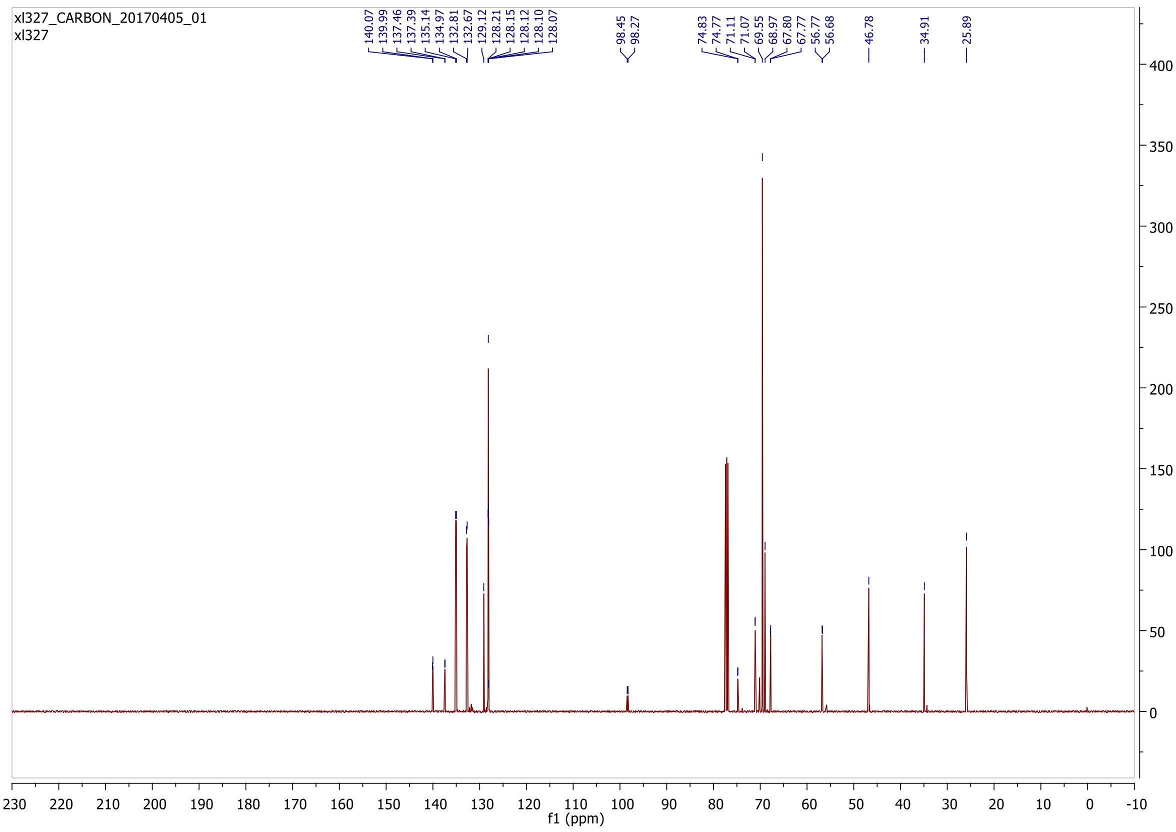

^{13}C NMR Spectrum of (*R*,*Rp*)-**15**

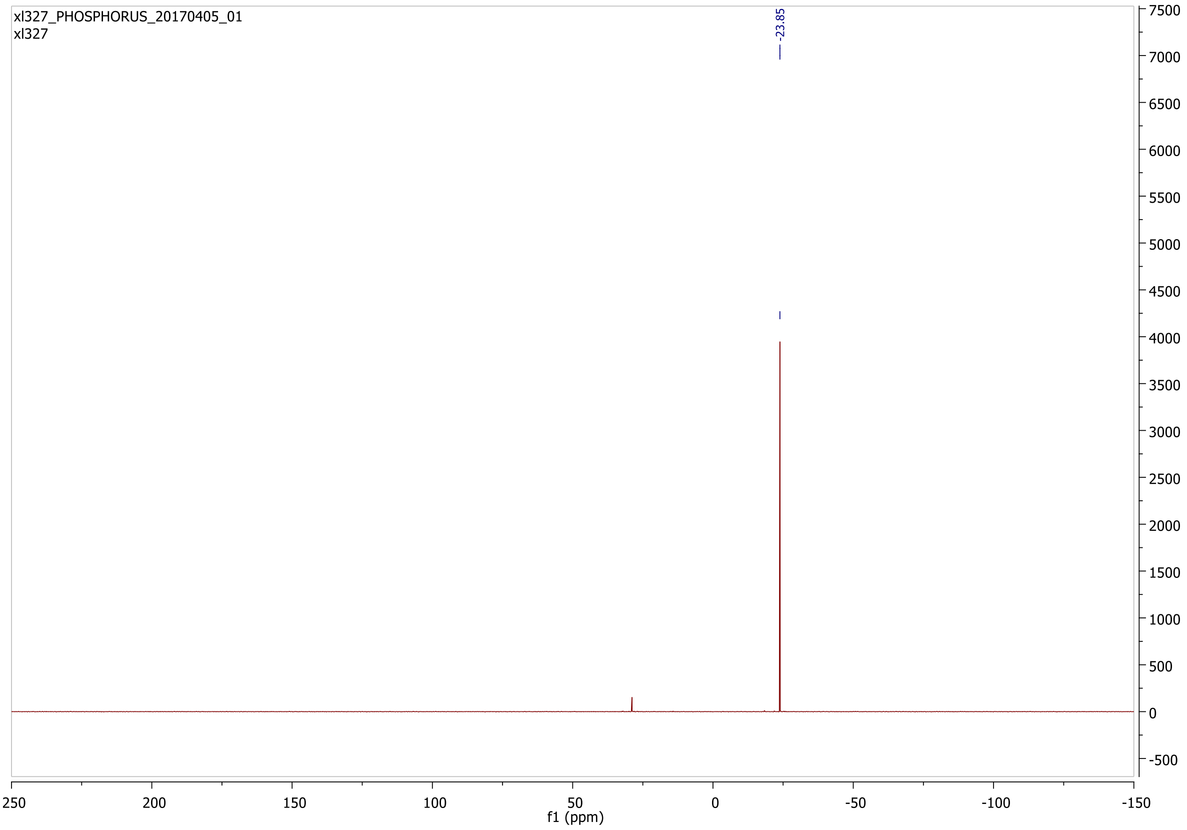

^{31}P NMR Spectrum of (*R*,*Rp*)-**15**

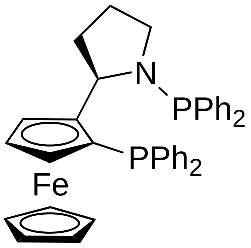

L1

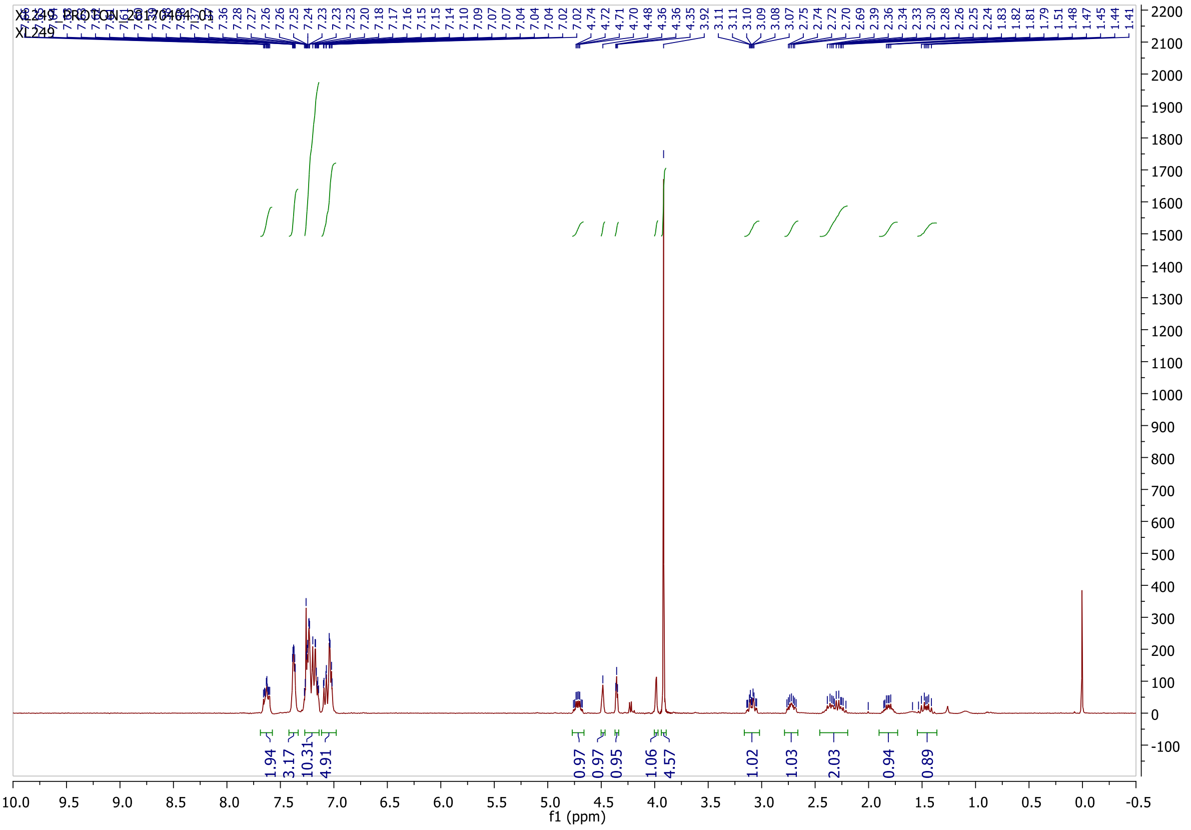

^{1}H NMR Spectrum of ligand **L1**

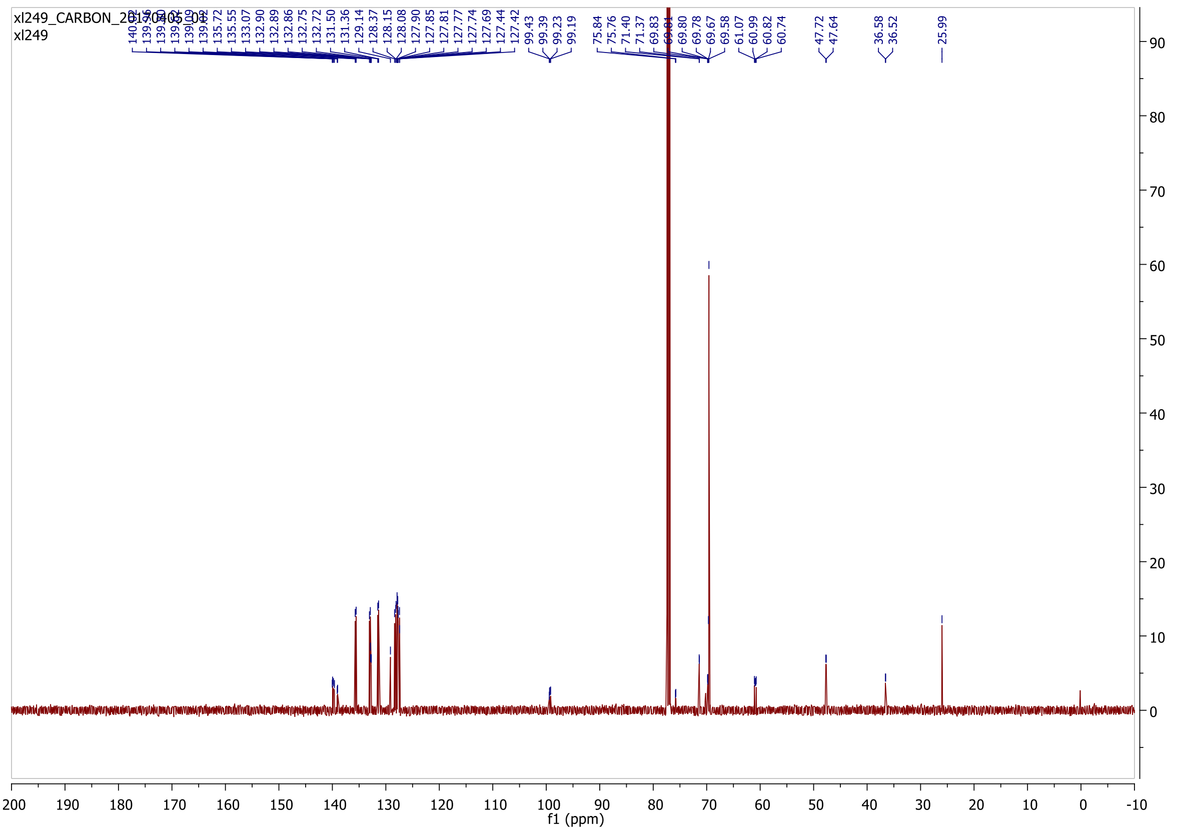

^{13}C NMR Spectrum of ligand **L1**

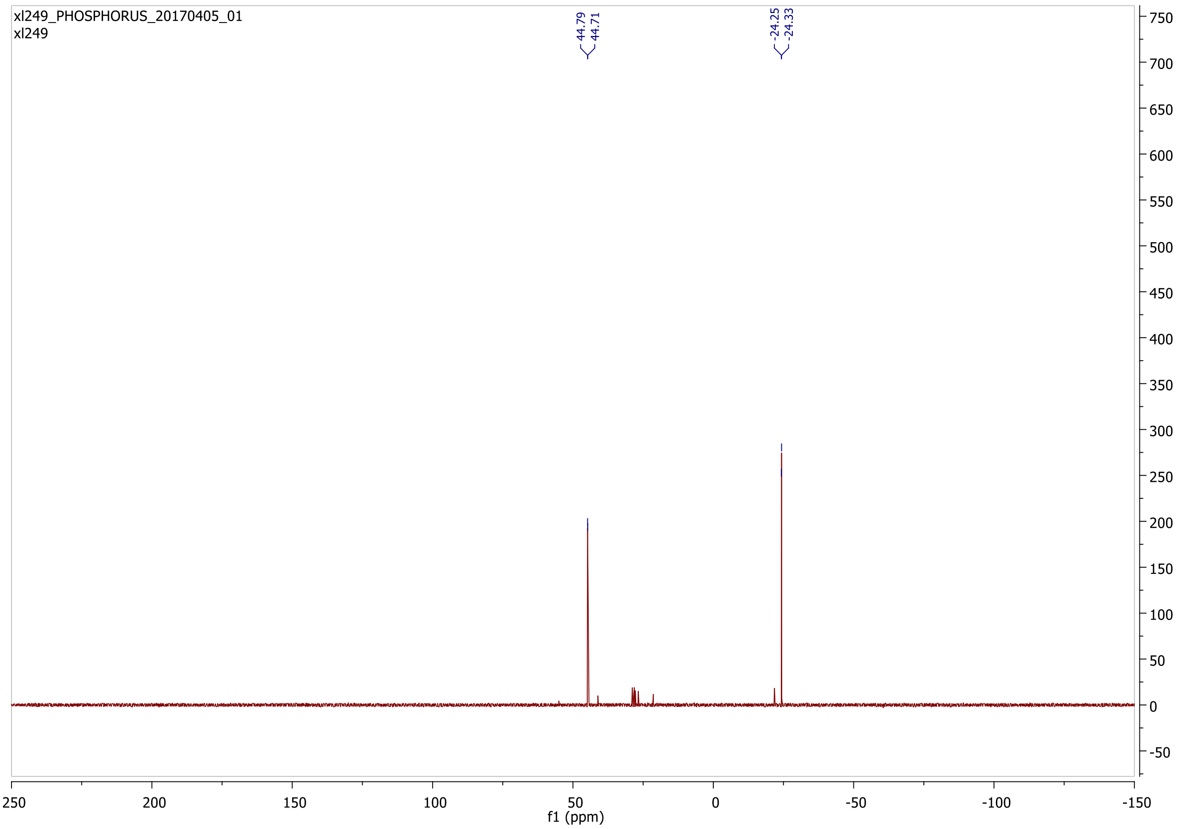

^{31}P NMR Spectrum of ligand **L1**

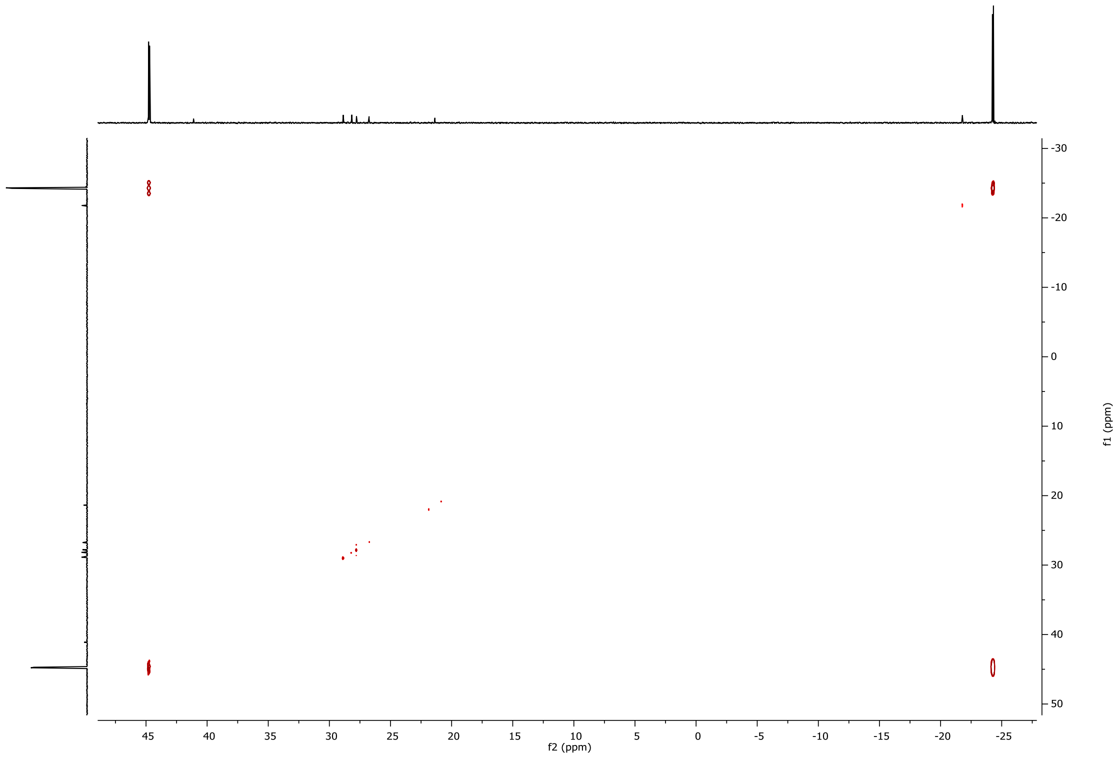

^{31}P-^{31}P COSY of Ligand **L1**

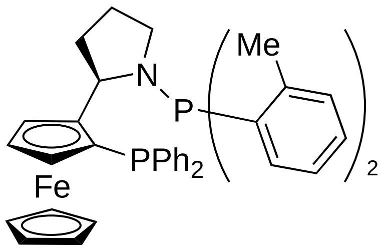

L2

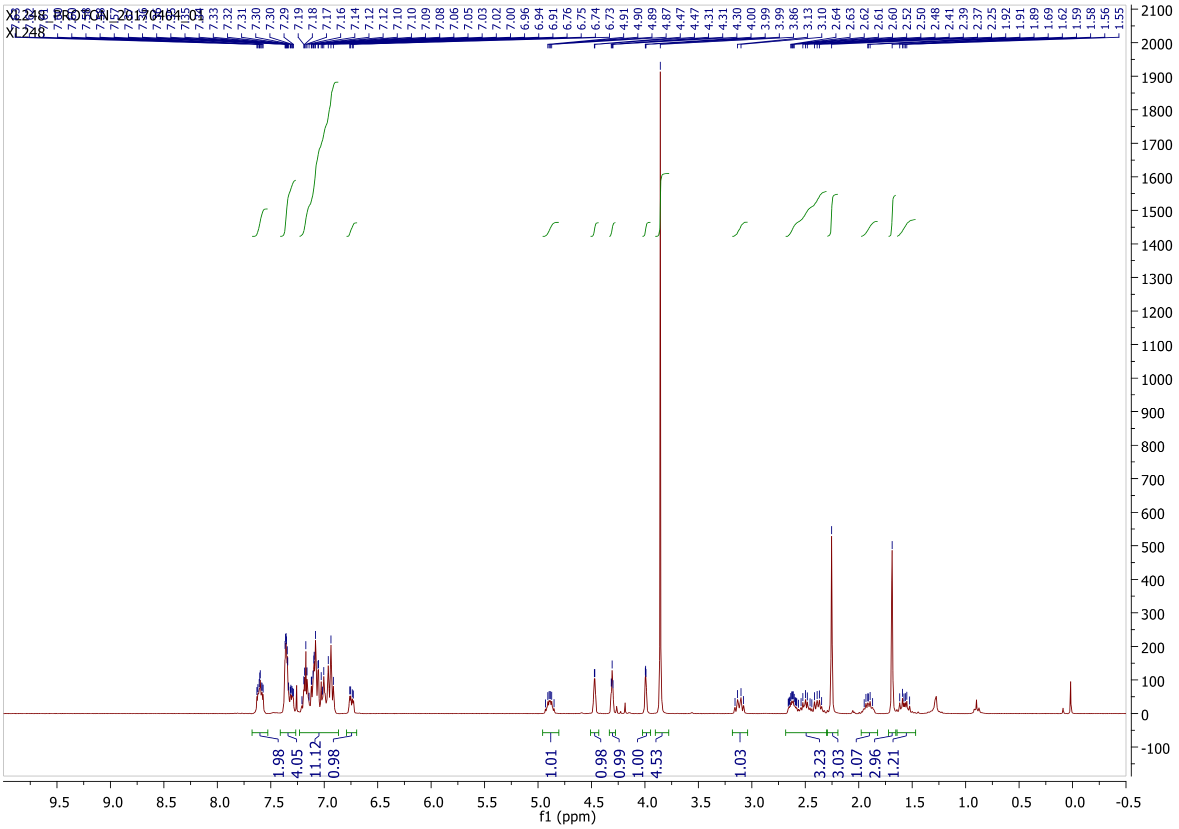

^{1}H NMR Spectrum of ligand **L2**

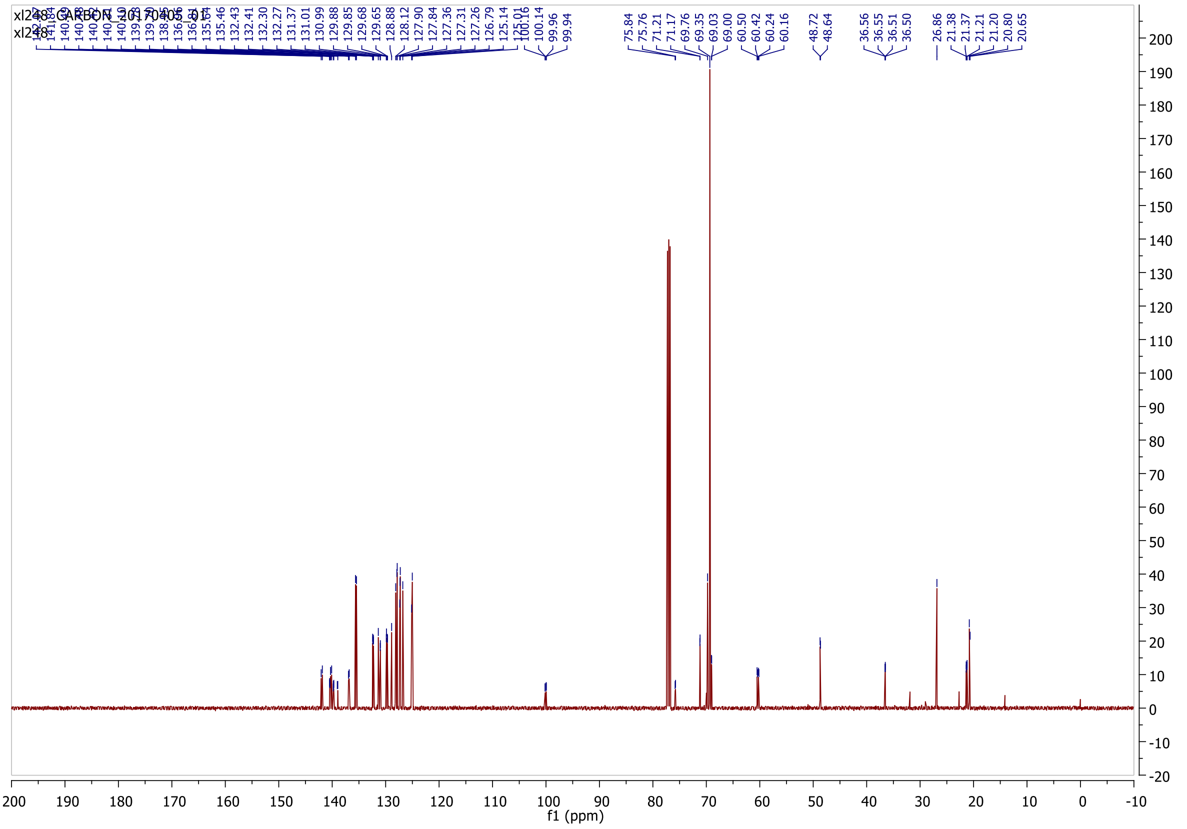

^{13}C NMR Spectrum of ligand **L2**

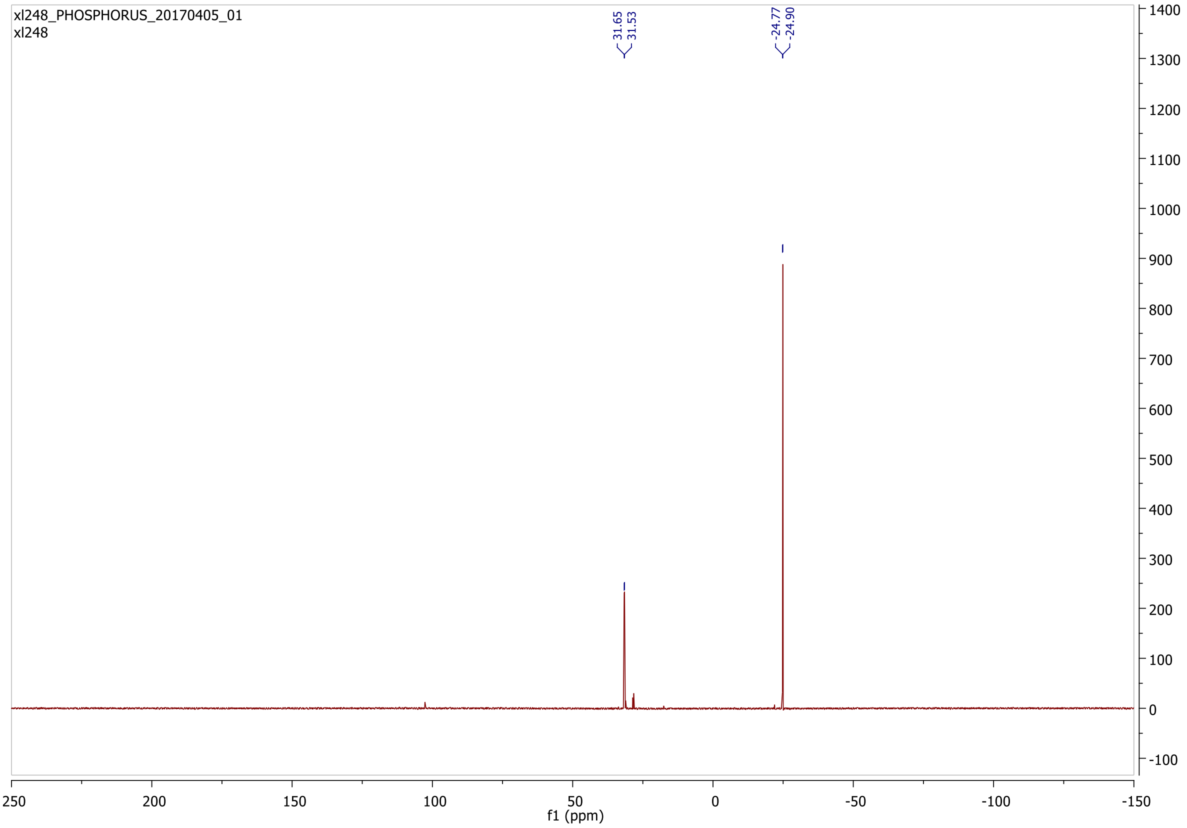

^{31}P NMR Spectrum of ligand **L2**

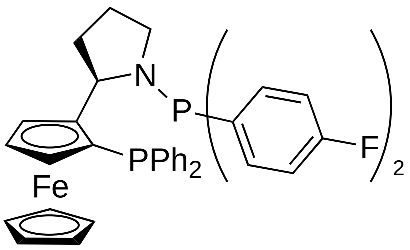

L3

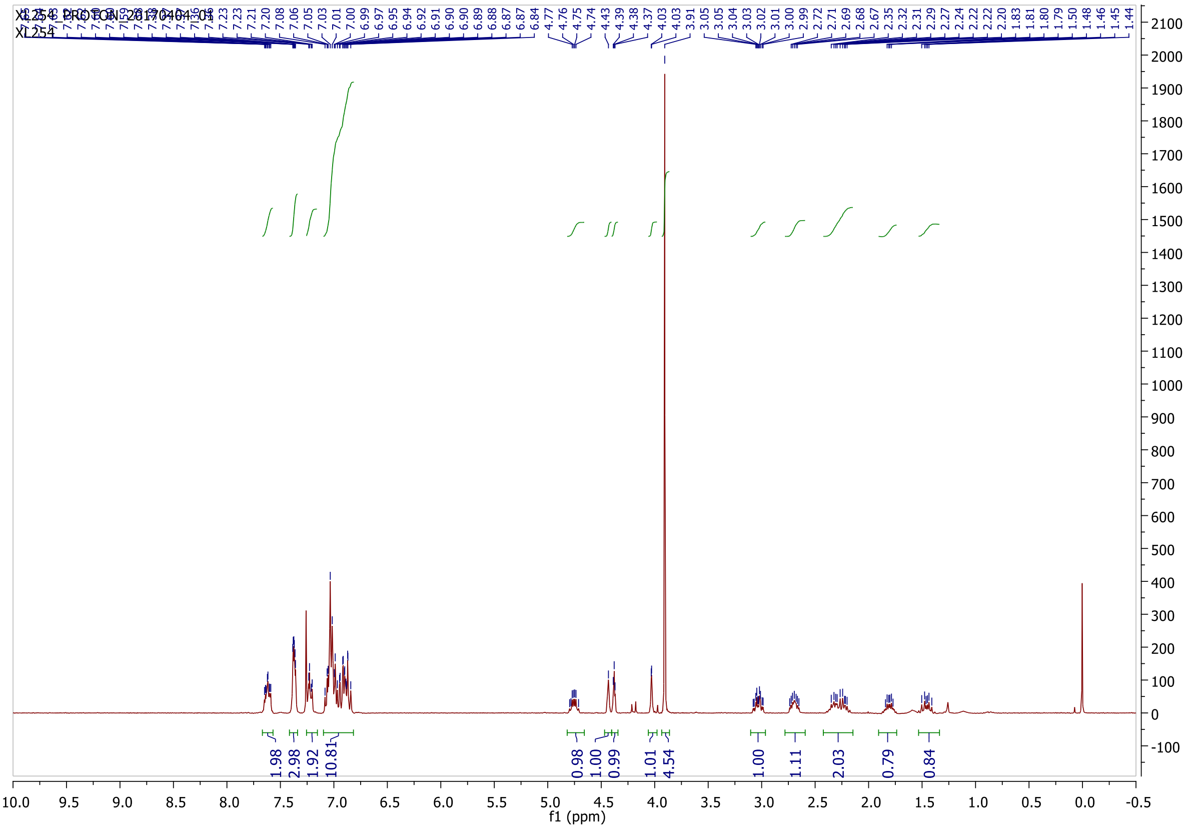

^{1}H NMR Spectrum of ligand **L3**

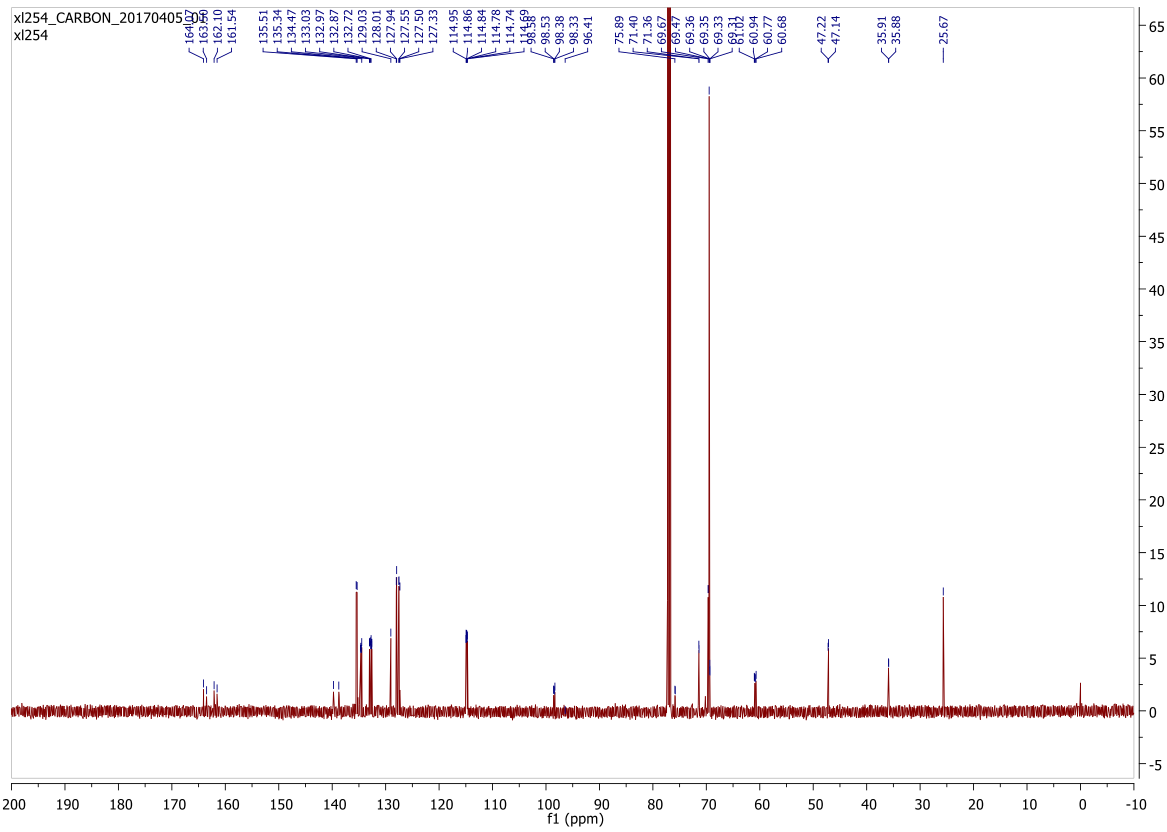

^{13}C NMR Spectrum of ligand **L3**

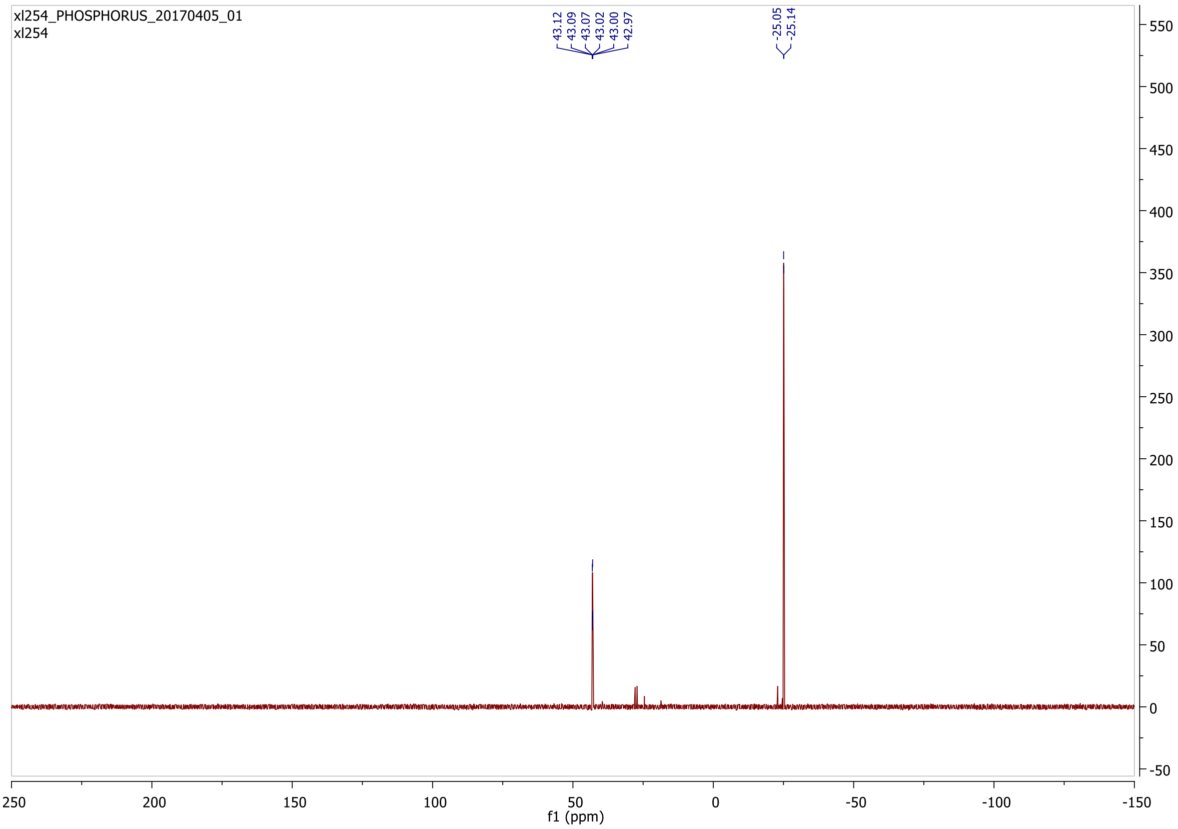

^{31}P NMR Spectrum of ligand **L3**

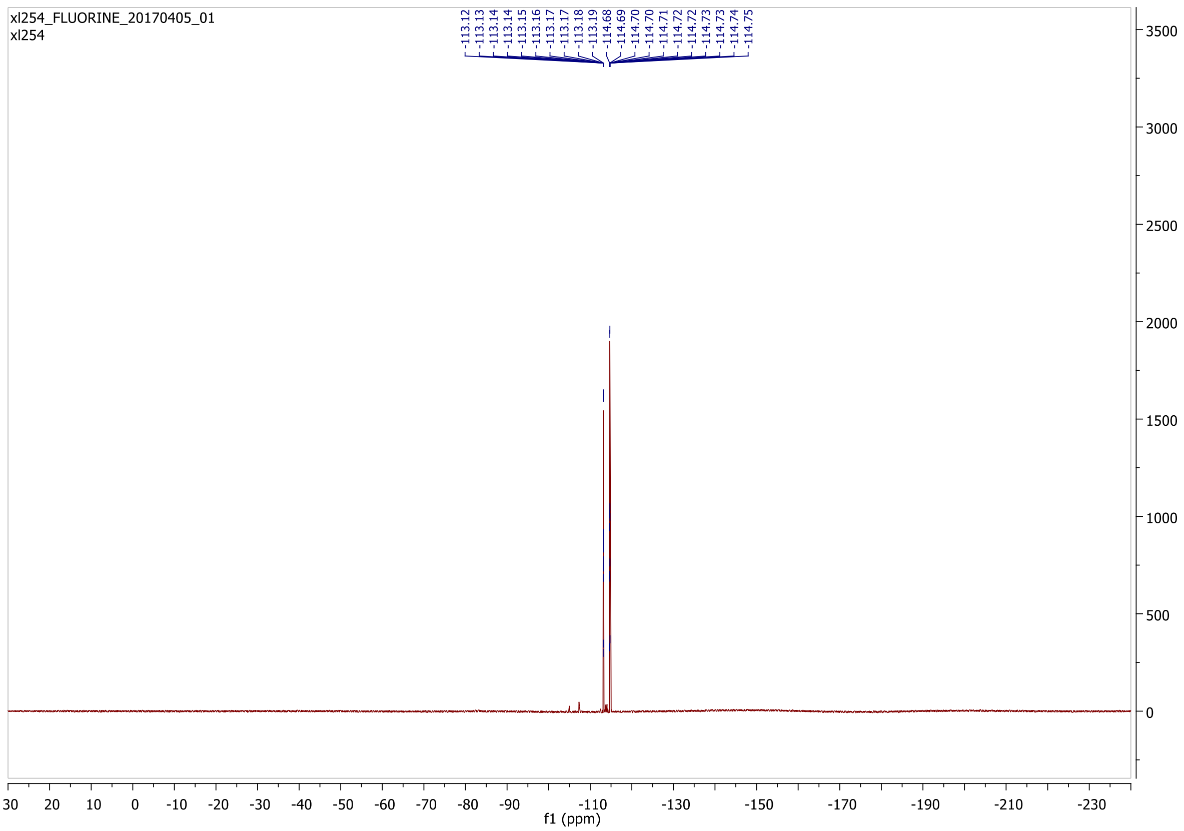

^{19}F NMR Spectrum of ligand **L3**

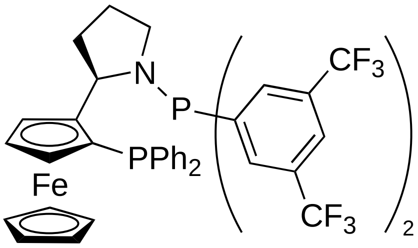

L4

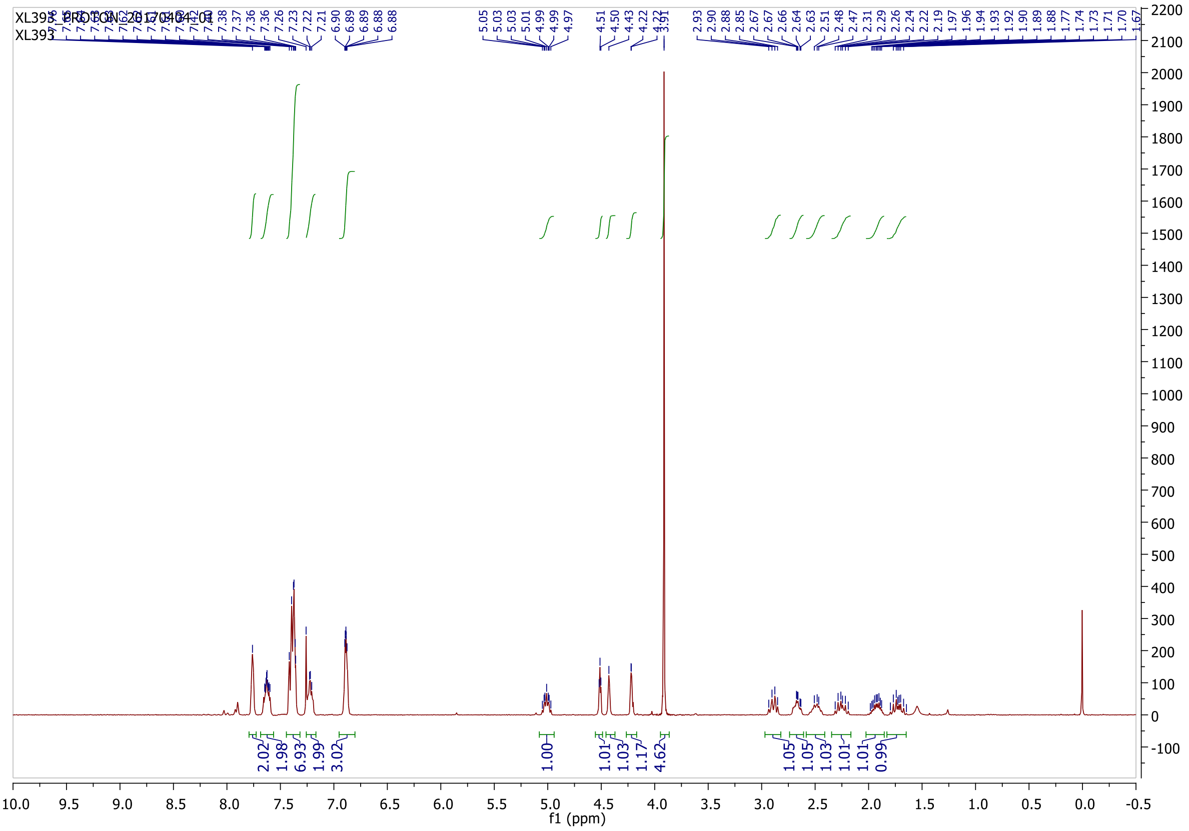

^{1}H NMR Spectrum of ligand **L4**

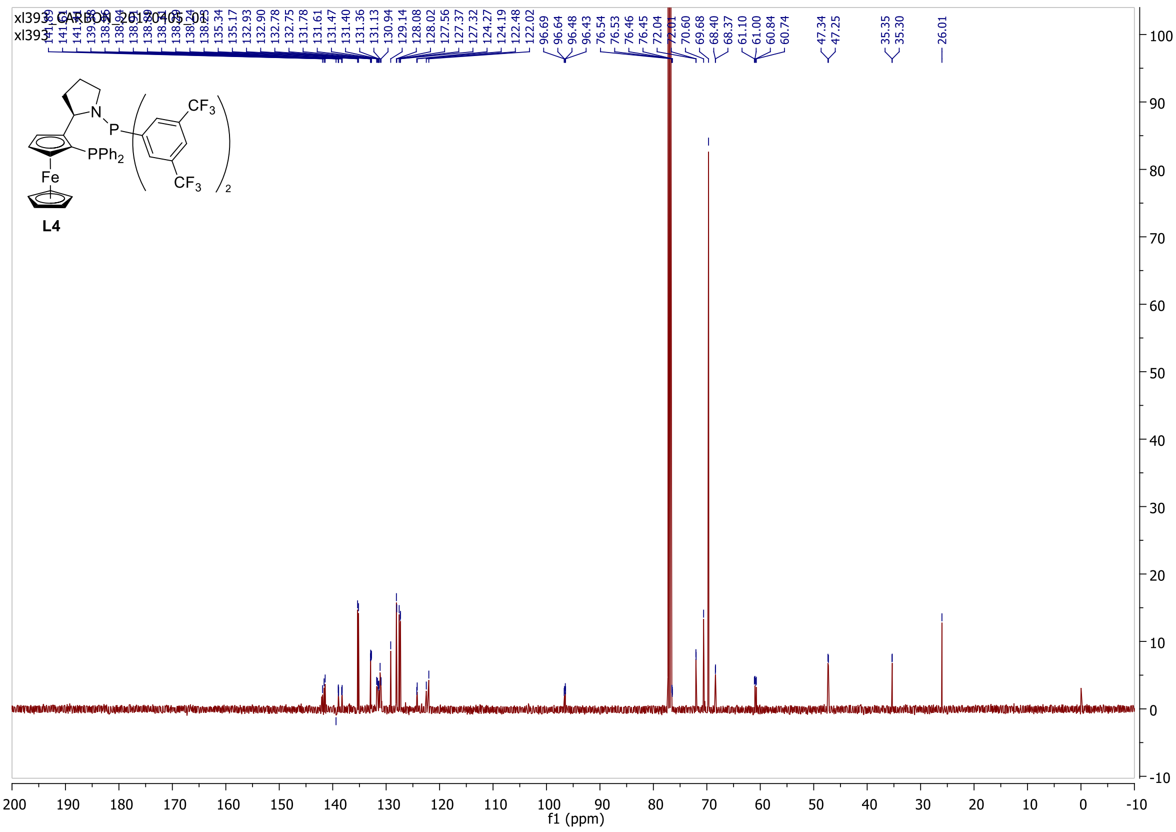

^{13}C NMR Spectrum of ligand **L4**

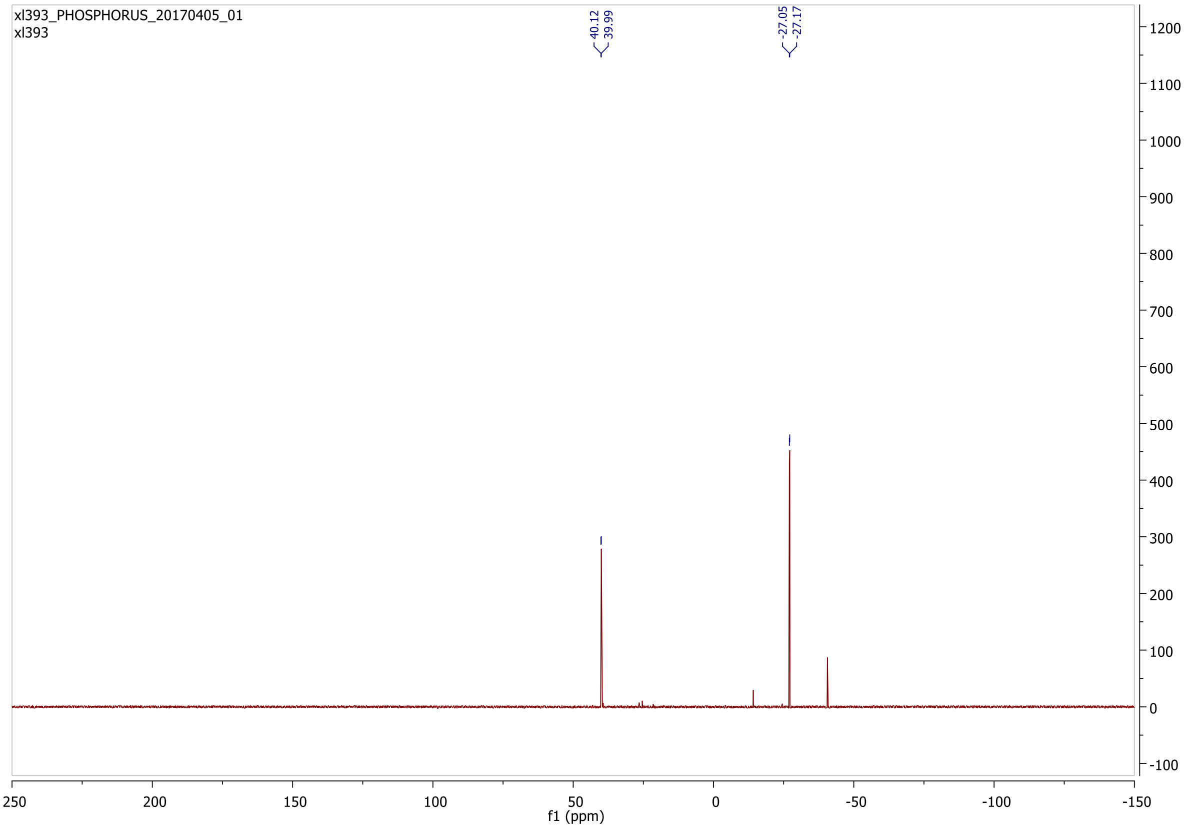

^{31}P NMR Spectrum of ligand **L4**

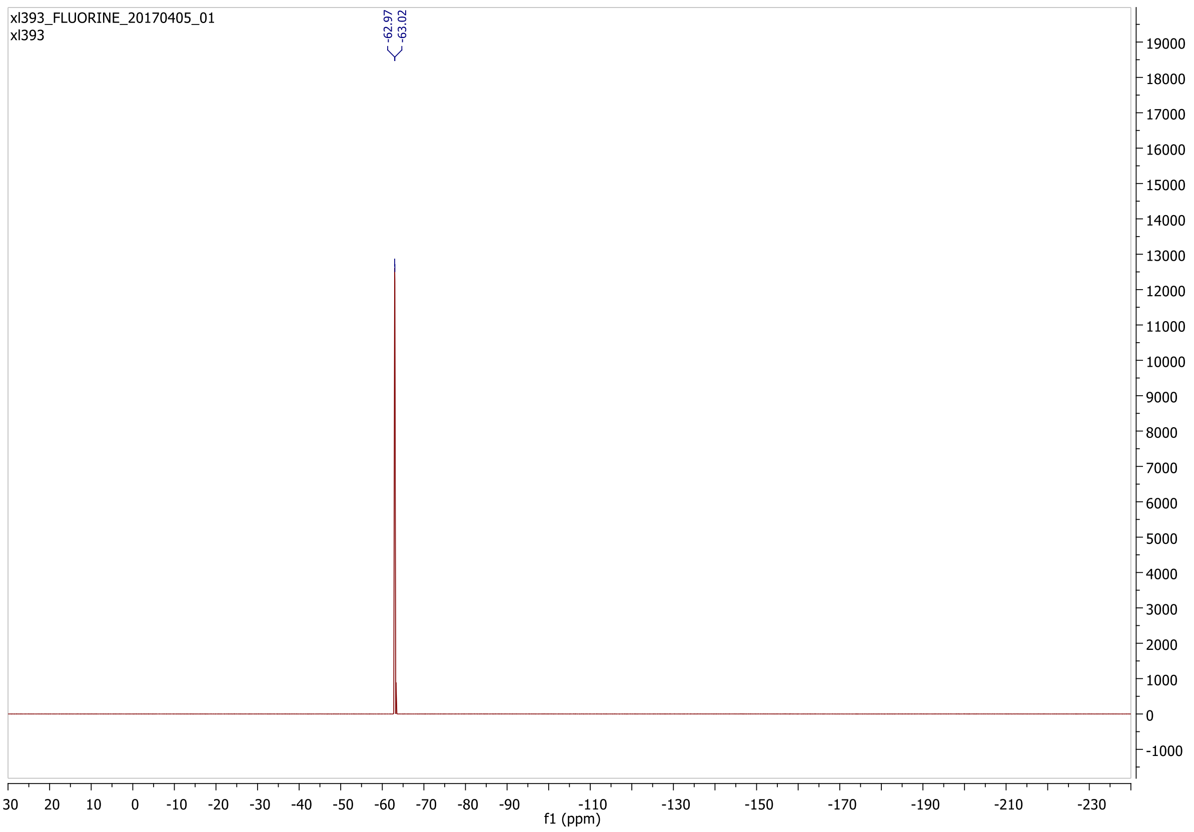

^{19}F NMR Spectrum of ligand **L4**

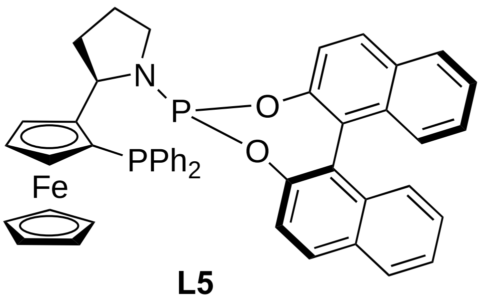

L5

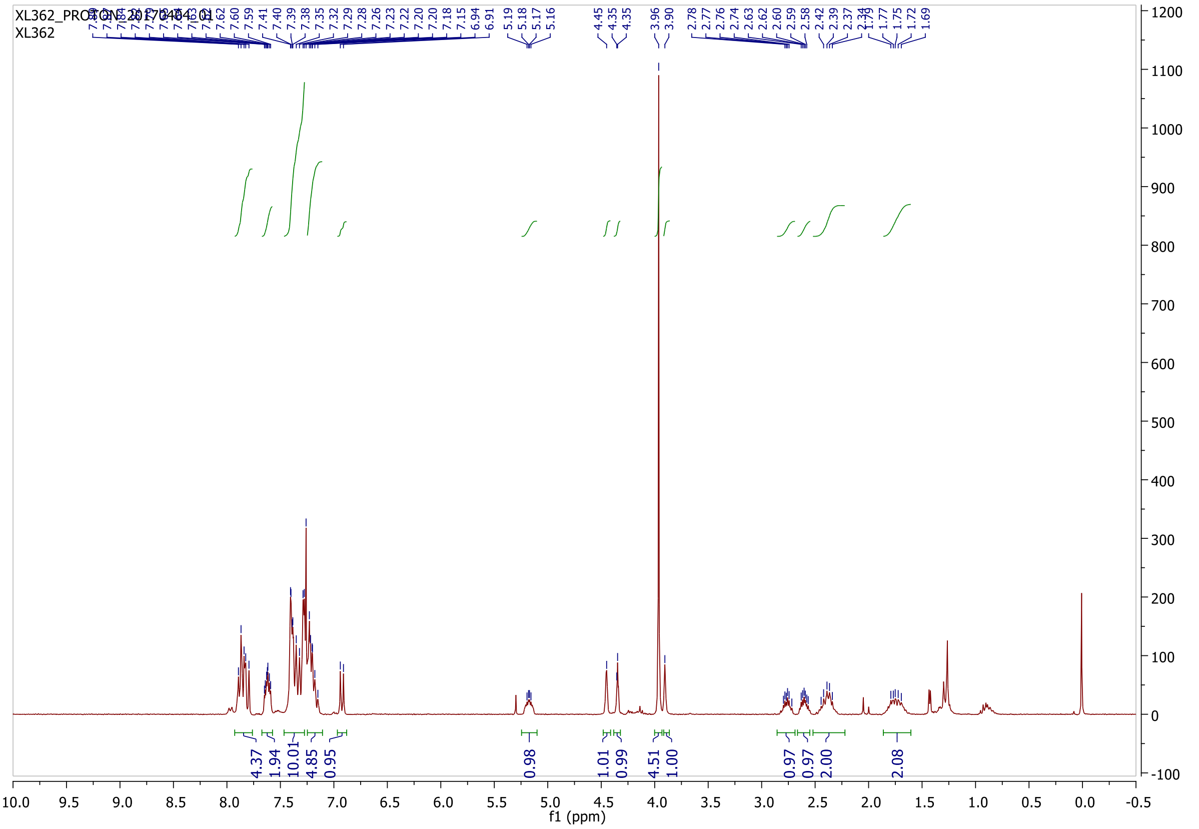

^{1}H NMR Spectrum of ligand **L5**

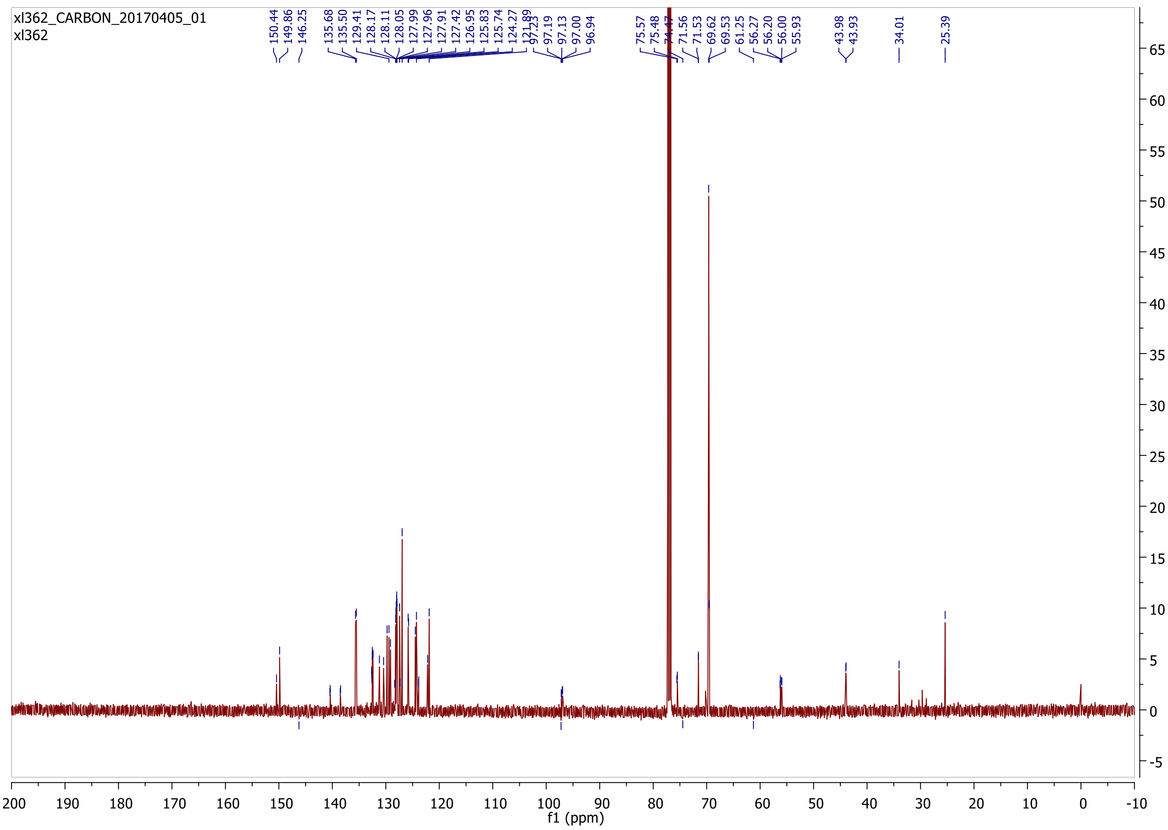

^{13}C NMR Spectrum of ligand **L5**

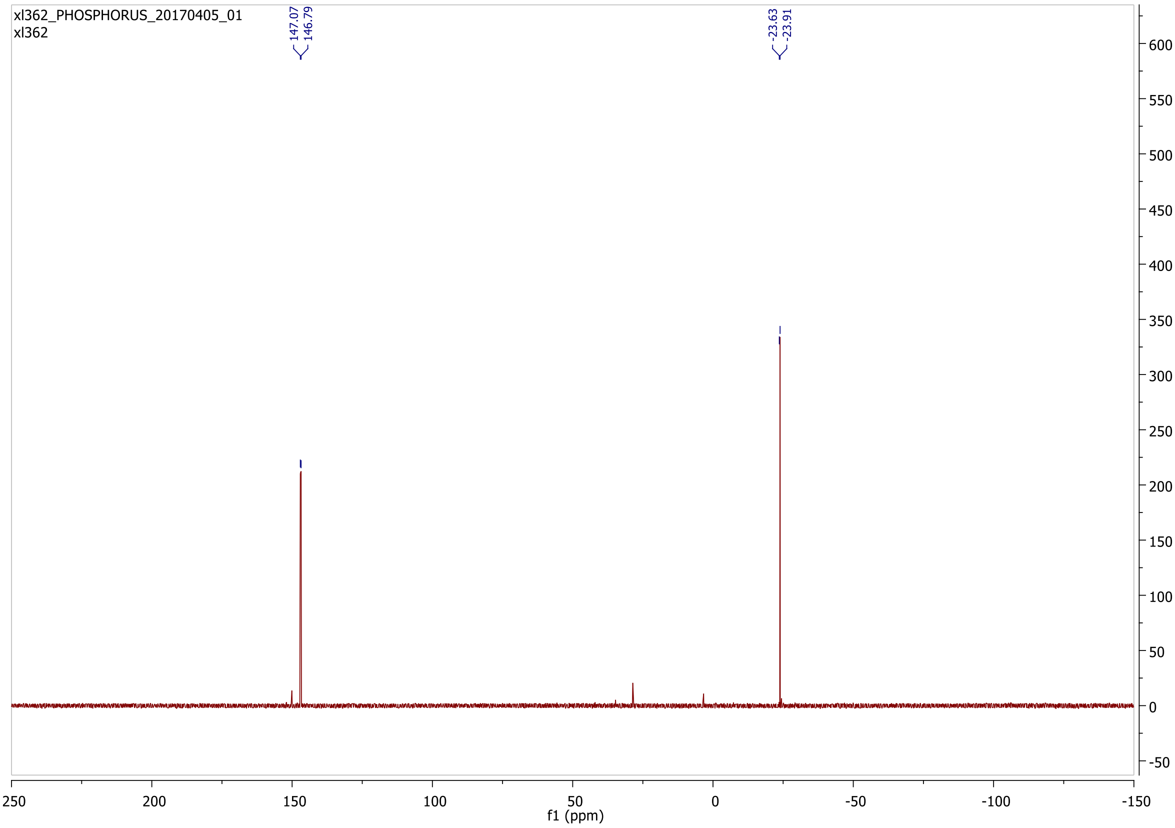

^{31}P NMR Spectrum of ligand **L5**

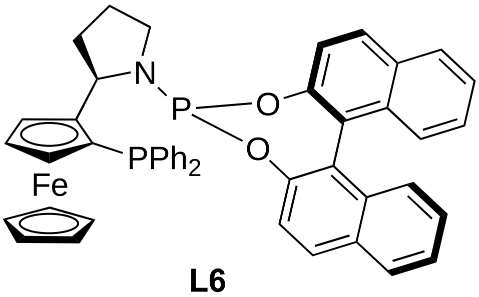

L6

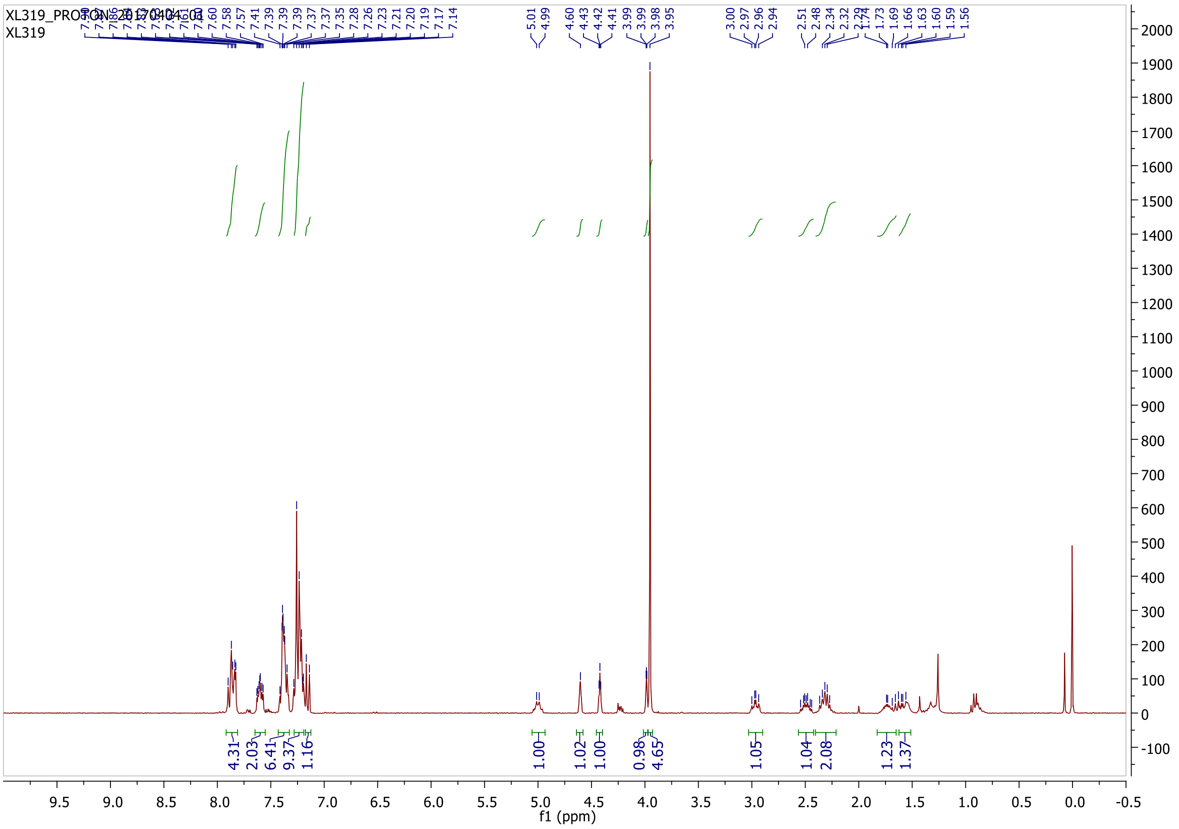

^{1}H NMR Spectrum of ligand **L6**

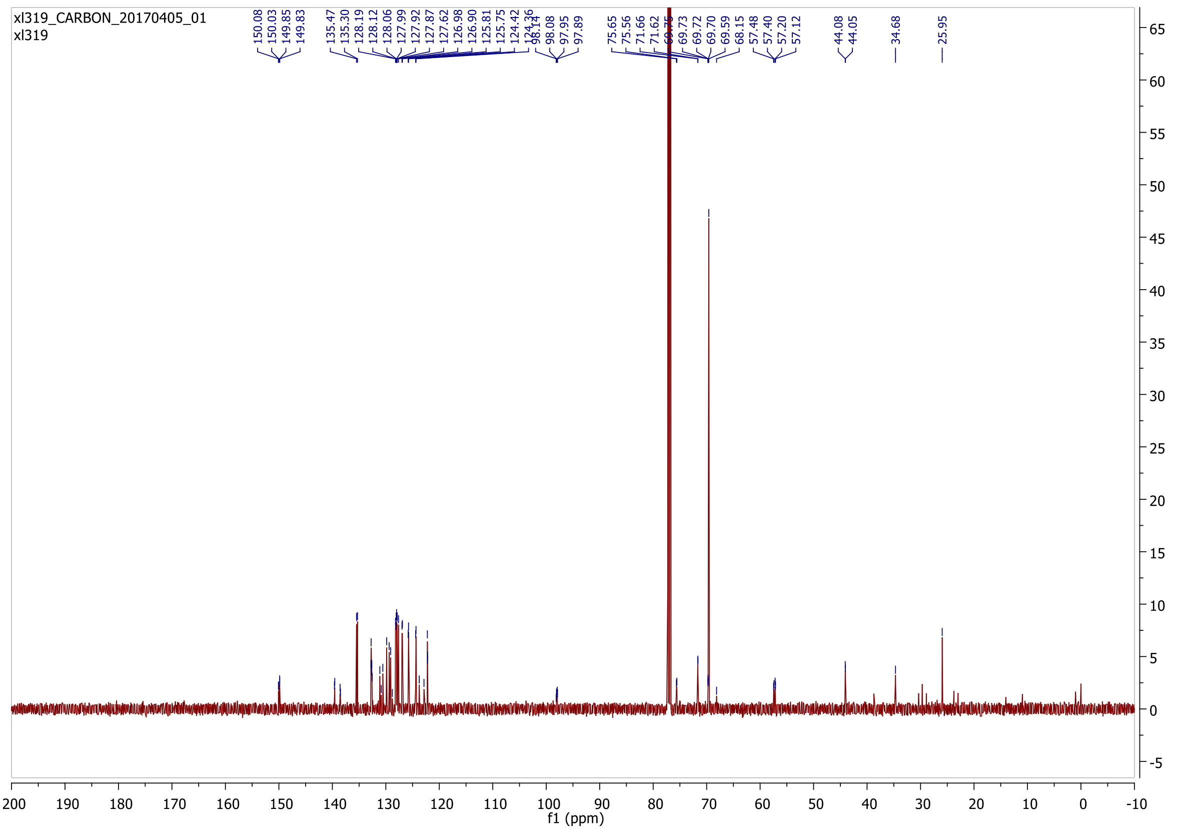

^{13}C NMR Spectrum of ligand **L6**

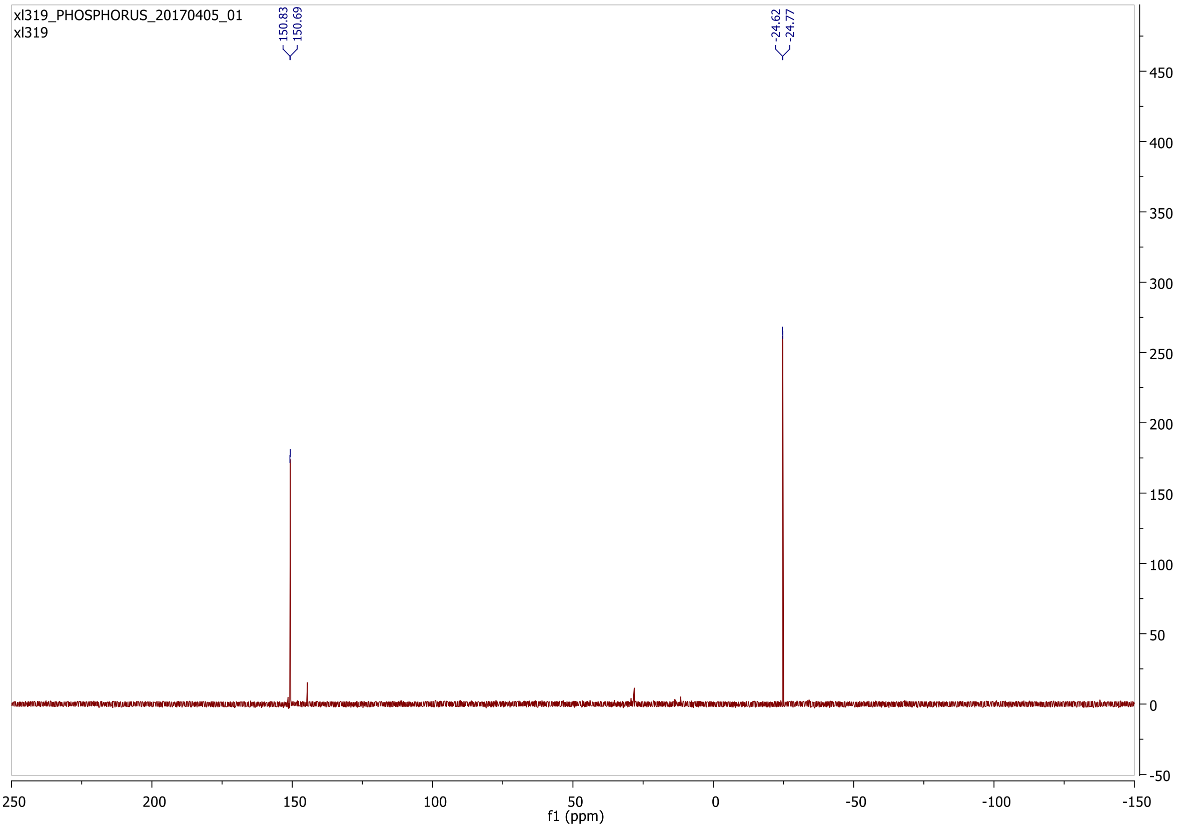

^{31}P NMR Spectrum of ligand **L6**

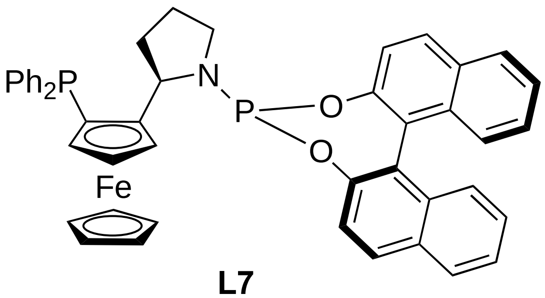

L7

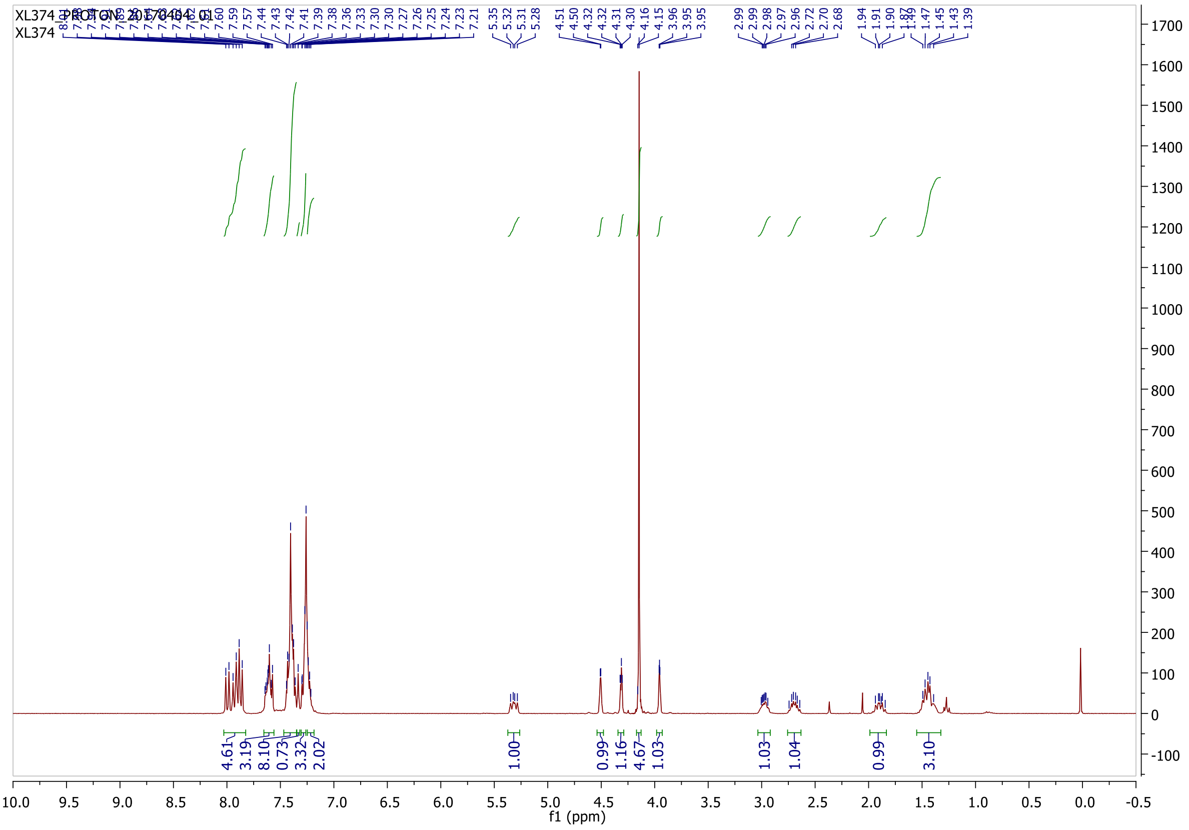

^{1}H NMR Spectrum of ligand **L7**

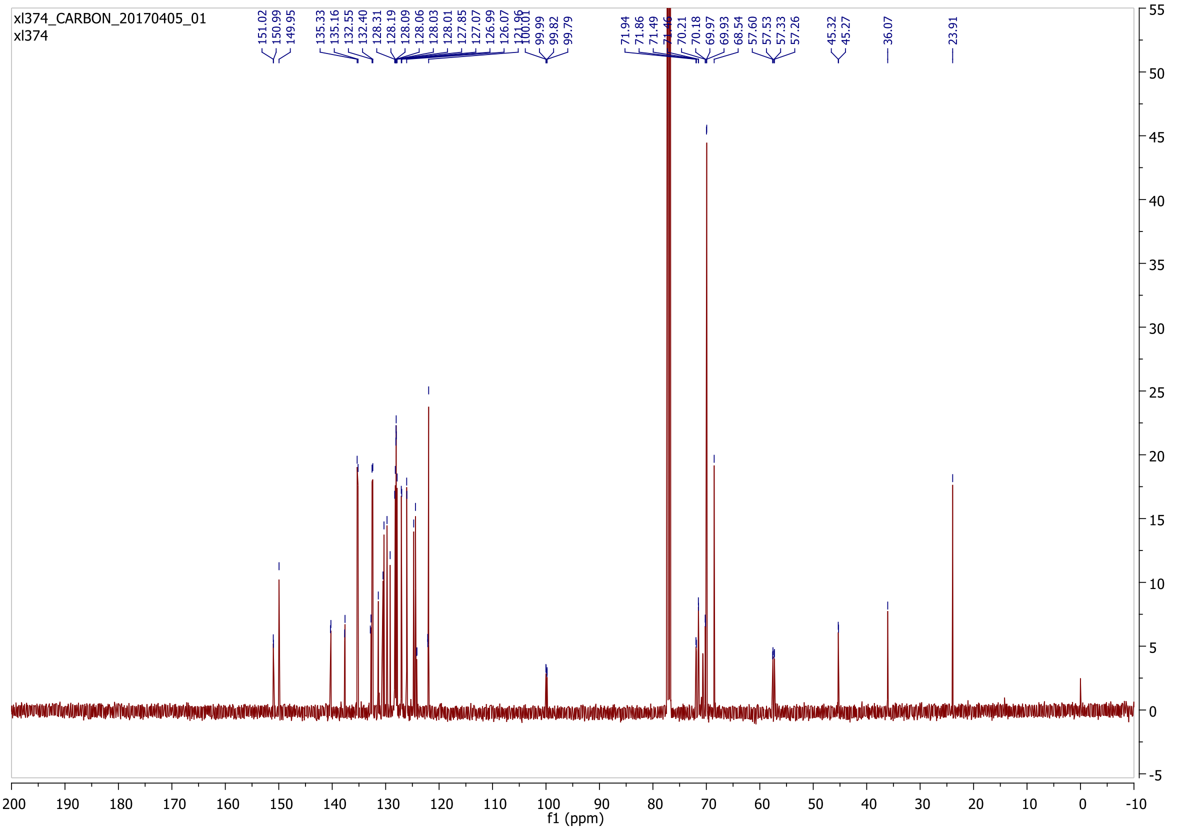

^{13}C NMR Spectrum of ligand **L7**

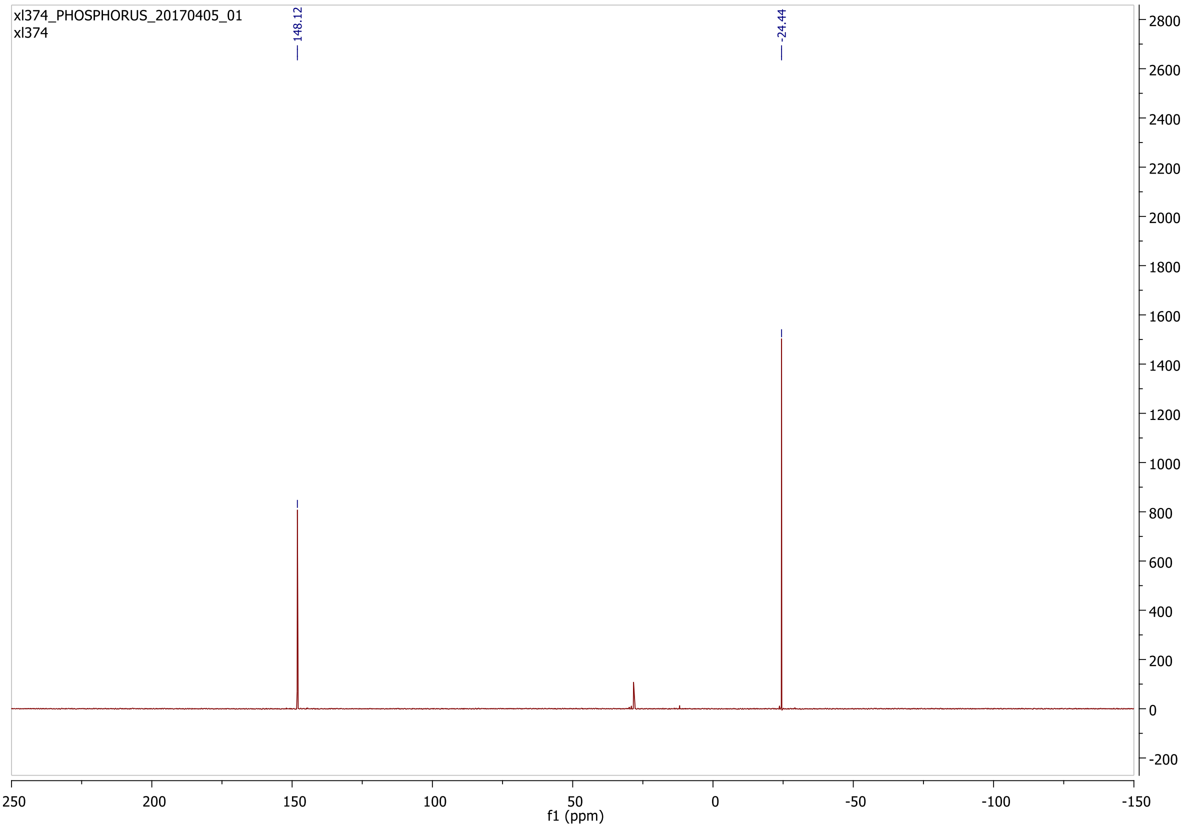

^{31}P NMR Spectrum of ligand **L7**

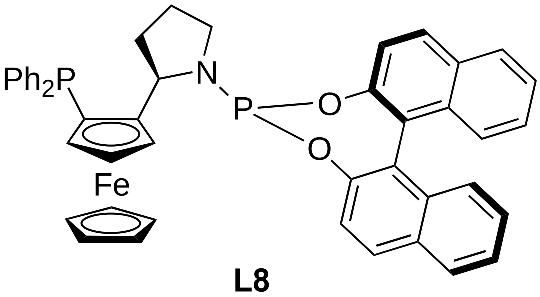

L8

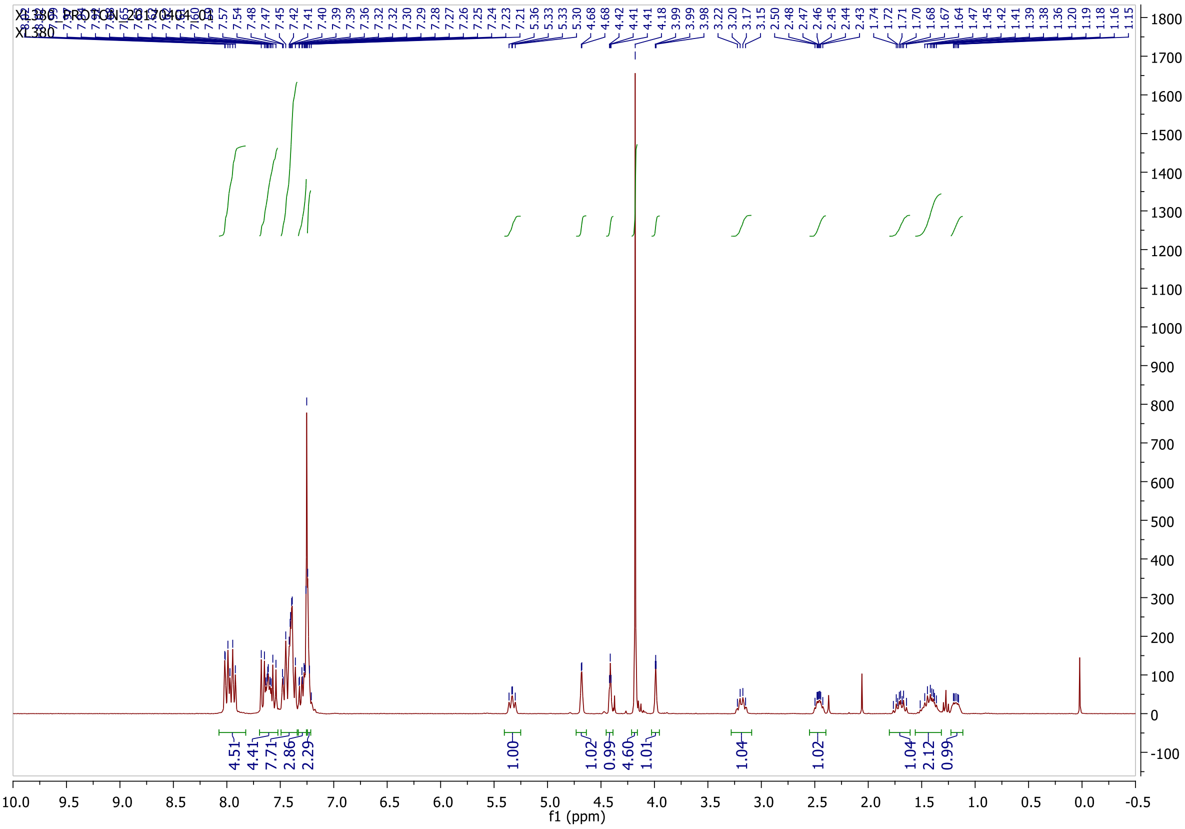

^{1}H NMR Spectrum of ligand **L8**

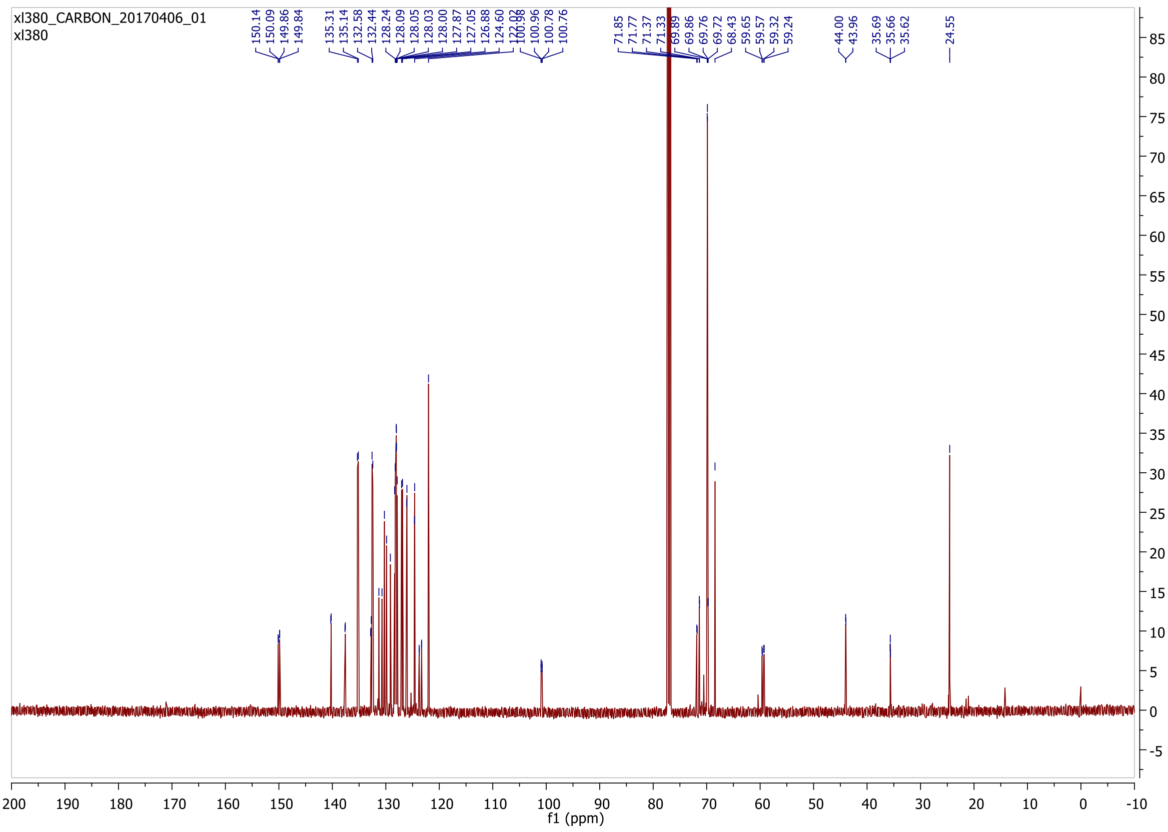

^{13}C NMR Spectrum of ligand **L8**

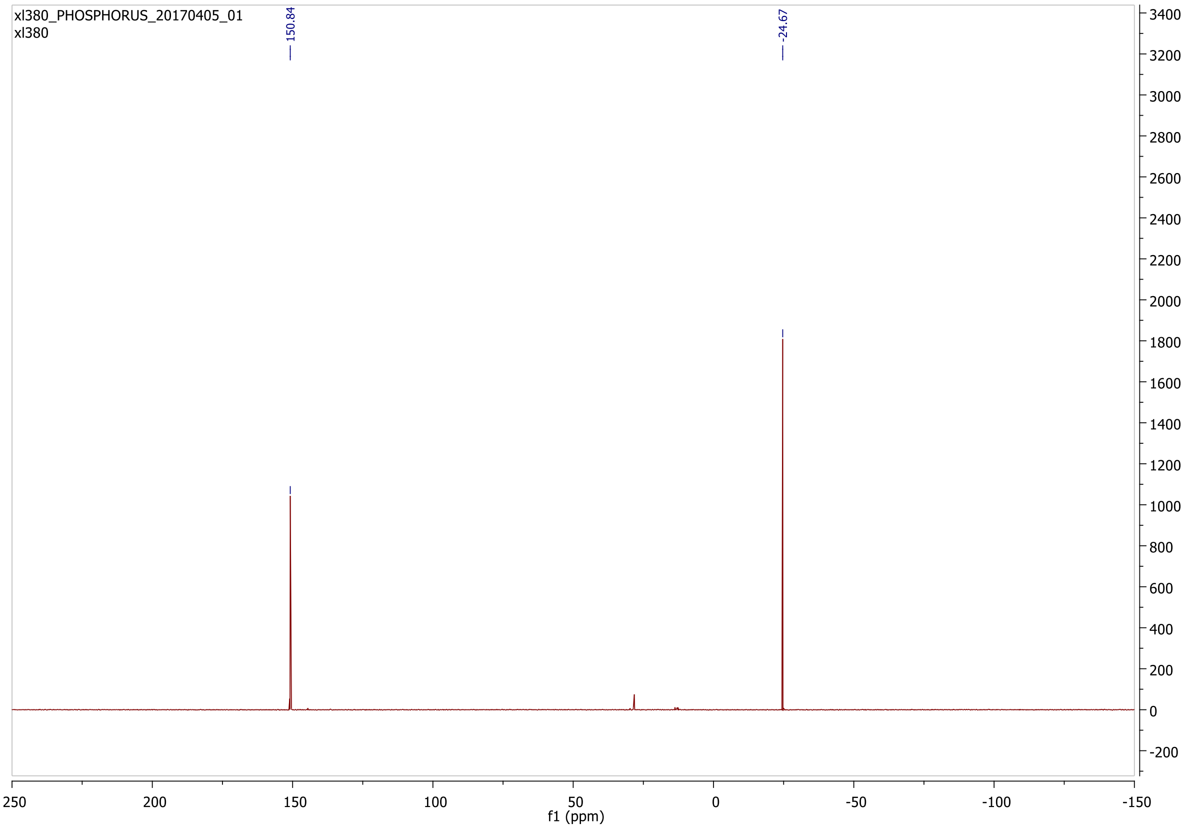

^{31}P NMR Spectrum of ligand **L8**

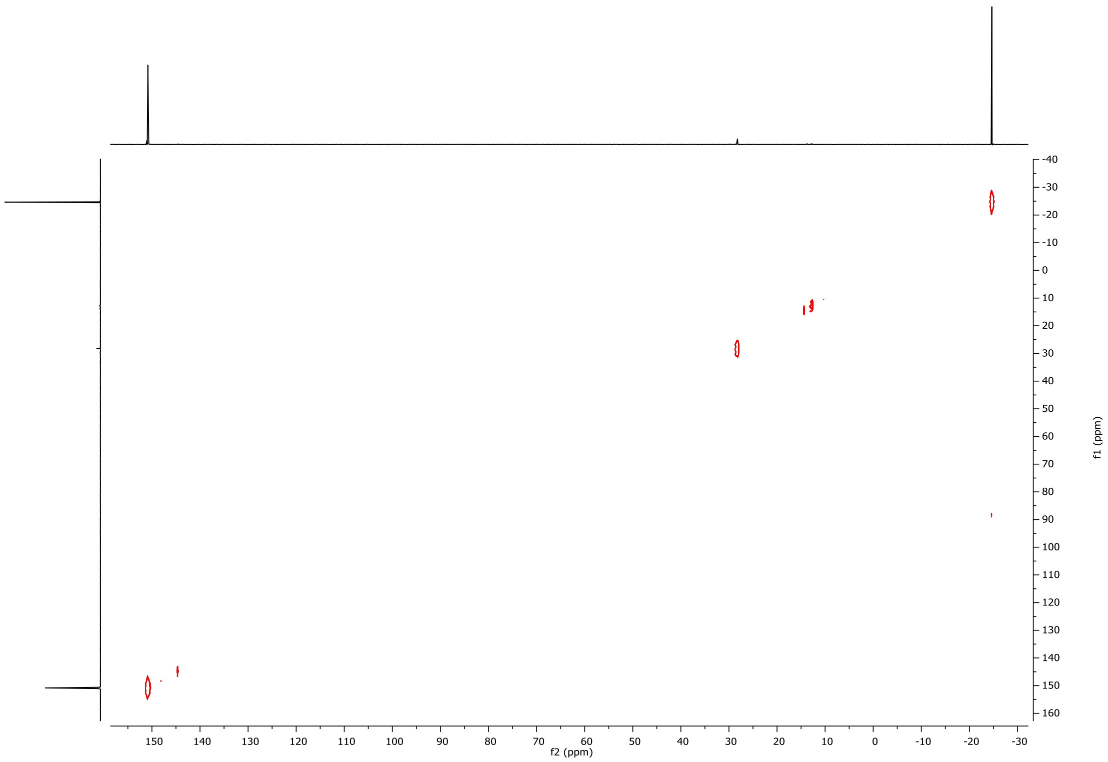

^{31}P-^{31}P COSY of ligand **L8**

X-Ray Crystallographic Structure of L5

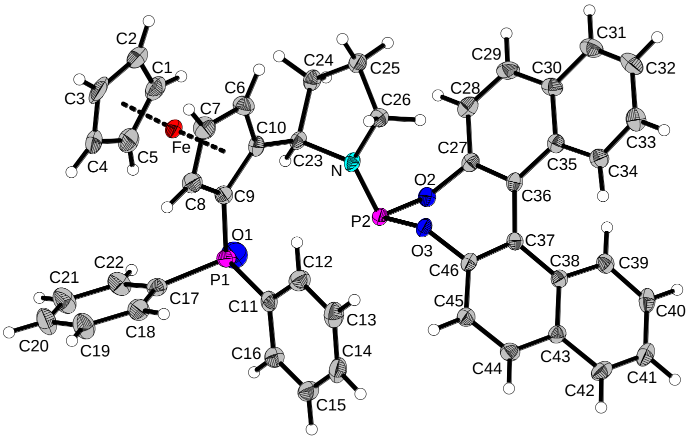

L5 (molecule; thermal ellipsoids are drawn on the 50% probability level; O1 15% occupied)

Crystal data and structure refinement for **L5**.

Identification code	gui53
Empirical formula	C46 H37 Fe N O2.15 P2
Formula weight	755.91
Temperature	100(2) K
Wavelength	1.54184 Å
Crystal system	Monoclinic
Space group	P 21
Unit cell dimensions	a = 8.0501(1) Å
	b = 15.2402(3) Å
	c = 14.8419(2) Å
Volume	1806.83(5) $Å^3$
Z	2
Density (calculated)	1.389 Mg/m^3
Absorption coefficient	4.510 mm^{-1}
F(000)	786.4
Crystal size	0.3073 x 0.2633 x 0.0342 mm^3
Theta range for data collection	4.17 to 65.19°.
Index ranges	-9<=h<=9, -17<=k<=17, -17<=l<=17
Reflections collected	14880
Independent reflections	6156 [R(int) = 0.0345]
Completeness to theta = 65.19°	99.8 %
Absorption correction	Analytical
Max. and min. transmission	0.860 and 0.397
Refinement method	Full-matrix least-squares on F^2
Data / restraints / parameters	6156 / 1 / 474
Goodness-of-fit on F^2	1.066
Final R indices [I>2sigma(I)]	R1 = 0.0355, wR2 = 0.0891
R indices (all data)	R1 = 0.0386, wR2 = 0.0906
Absolute structure parameter	-0.017(3)
Largest diff. peak and hole	0.247 and -0.340 e.$Å^{-3}$
